# Development of a food allergy education resource for primary care physicians

**DOI:** 10.1186/1472-6920-8-45

**Published:** 2008-09-30

**Authors:** Joyce E Yu, Arvind Kumar, Christine Bruhn, Suzanne S Teuber, Scott H Sicherer

**Affiliations:** 1 Division of Allergy and Immunology, Department of Pediatrics, Mount Sinai School of Medicine, New York, NY, USA; 2 Division of Rheumatology, Allergy, and Clinical Immunology, Department of Internal Medicine, University of California, Davis, School of Medicine, Davis, CA, USA

## Abstract

**Background:**

Food allergy is estimated to affect 3–4% of adults in the US, but there are limited educational resources for primary care physicians. The goal of this study was to develop and pilot a food allergy educational resource based upon a needs survey of non-allergist healthcare providers.

**Methods:**

A survey was undertaken to identify educational needs and preferences for providers, with a focus on physicians caring for adults and teenagers, including emergency medicine providers. The results of the survey were used to develop a teaching program that was subsequently piloted on primary care and emergency medicine physicians. Knowledge base tests and satisfaction surveys were administered to determine the effectiveness of the educational program.

**Results:**

Eighty-two physicians (response rate, 65%) completed the needs assessment survey. Areas of deficiency and educational needs identified included: identification of potentially life-threatening food allergies, food allergy diagnosis, and education of patients about treatment (food avoidance and epinephrine use). Small group, on-site training was the most requested mode of education. A slide set and narrative were developed to address the identified needs. Twenty-six separately enrolled participants were administered the teaching set. Pre-post knowledge base scores increased from a mean of 38% correct to 64% correct (p < 0.001). Ability to correctly demonstrate the use of epinephrine self injectors increased significantly. Nearly all participants (>95%) indicated that the teaching module increased their comfort with recognition and management of food allergy.

**Conclusion:**

Our pilot food allergy program, developed based upon needs assessments, showed strong participant satisfaction and educational value.

## Background

It is estimated that 3–4% of adults have food allergy, and that 20% of adults avoid a food based upon a perceived allergy [1,2]. Teenagers and young adults appear to be at highest risk for fatal food allergies [3,4]. Food allergy is apparently increasing, at least for allergy to peanuts [5,6]. Primary care and emergency medicine healthcare providers are called upon to diagnose and treat these patients, but previous studies indicate a significant deficit in appropriate diagnosis and treatment. A national survey of pediatricians identified knowledge base deficits where nearly half of the respondents did not properly recognize and treat food-induced anaphylaxis [7]. A study by Wang et al. similarly identified that half of the respondents failed to treat a hypothetical case of anaphylaxis with the appropriate dose of epinephrine, and more than a third of respondents selected an inadequate observation period following a reaction [8]. In addition, a majority of respondents were unable to identify risk factors for anaphylaxis. A study on the ability of pediatricians to correctly demonstrate epinephrine self-injector use revealed that the majority of pediatricians were not able to correctly use the epinephrine devices [9]. Finally, a study across 21 emergency rooms revealed deficits in treatment of persons with food allergy including failure to provide referral, training on avoidance, and treatment in the emergency department [10].

In an effort to improve management of food allergy and anaphylaxis by non-allergist healthcare providers, we sought to identify the educational needs of these stakeholders and create teaching materials to effectively address these needs. We focused upon primary care and emergency medicine physicians likely to care for those at highest risk for fatal anaphylaxis (teenagers and young adults). We created and administered a needs assessment survey and used those results to form an educational program that was further piloted to determine efficacy and satisfaction.

## Methods

### Needs assessment survey

The investigators created a 23-item, structured, written questionnaire (refined by administration to and feedback from 3 allergists and 3 primary care physicians) that collected information on physician demographics, practice type, prior exposure to food allergy education, and self-reported knowledge of food allergy and anaphylaxis management. Individual feedback regarding specific educational needs in the area of food allergy and learning style preferences was also obtained.

A convenience sample of physicians with different specialties was obtained by distributing surveys in person during departmental grand rounds and staff meetings, and by interoffice mail (in the case of emergency medicine physicians in New York). Responses were collected anonymously. All studies were approved by the Institutional Review Board at Mount Sinai and University of California, Davis.

### Educational program design and testing

Based upon the indicated learning preferences, educational material comprised of a PowerPoint slide set and narrative and a self-injectable epinephrine demonstration was developed and written by board-certified allergists specializing in food allergy [SS, ST] for a target audience of non-allergist healthcare providers. The slide set and self-injectable epinephrine demonstration are provided in Additional data file 1. The pre-scripted didactic-style presentation was based on guidelines for anaphylaxis and food allergy established by the American Academy of Asthma, Allergy and Immunology and included a comprehensive review of clinical food allergy with an emphasis on previously identified knowledge deficits and needs [11-14]. A live demonstration of how to utilize the self-injectable epinephrine devices, Epipen^® ^(Dey, Napa, CA) and Twinject^® ^(Verus, San Diego, CA), according to the manufacturers' guidelines was integrated into the discussion on anaphylaxis treatment. The slide set concluded with a number of interactive clinical case scenarios which highlighted the main concepts. The information was presented in an hour-long session with individual participants, who were administered a written knowledge pretest and post-test and were graded on their use of epinephrine self-injectors pre- and post-presentation. These participants were solicited primarily in person as a convenience sample from various clinical settings to obtain representation from several specialties. These participants were paid 100 US dollars to compensate for their time. The written test, which consisted of 10 single-best-answer, multiple-choice questions, was reviewed by healthcare providers caring for food allergy patients and non-allergist primary care physicians and revised to optimize question clarity and establish an appropriate difficulty level. A satisfaction survey (5 point Likert scale) was administered after the presentation. The steps in administering the Epipen^® ^autoinjector were scored in the order of use according to the instructions supplied by the manufacturer: 1) recognizing the device; 2) removing the cap; 3) selecting the appropriate body site; 4) pressing the correct end of the device to the body; 5) pressing to activate; 6) holding in place for several seconds. The steps in administering the two consecutive doses of the Twinject^® ^autoinjector were also scored in the order of use according to the instruction supplied by the manufacturer. The steps scored for the first injection were as follows: 1) recognizing the device; 2) removing the cap; 3) selecting the appropriate body site; 4) pressing the correct end of the device to the body; 5) pressing to activate; 6) holding in place for several seconds. The steps scored for the second injection were as follows: 1) opening the device for the inner syringe; 2) removing the safety ring; 3) injecting into the appropriate body site.

### Statistical analyses

Statistical analysis was performed using GraphPad Prism 4.0 software (San Diego, CA). Descriptive statistics are presented. Categorical variables were evaluated by the χ^2 ^test; test scores and epinephrine demonstration skill scores were analyzed using the Wilcoxon signed rank test. A p <0.05 was considered statistically significant.

## Results

### Needs assessment

We enrolled a total of 82 participants representing a convenience sample of board-certified primary care and emergency medicine physicians affiliated with the Mount Sinai Medical Center, New York, NY (49%) and the University of California, Davis Medical Center, Davis, CA (51%) who potentially care for teenagers and adults with food allergy. Response rates varied by venue: 100% from staff meetings (46 subjects), 49% from conferences/rounds (27 participants), and 36% (9 participants) from interoffice mailings (emergency medicine physicians in New York). The median number of years in clinical practice was 10 years (1–53 years). Table 1 summarizes the physician demographic characteristics. Nearly all physicians (98%) indicated that they care for patients with food allergies with half of these physicians treating 10 or more patients annually. The majority (80%) indicated that they had treated patients during an acute food-induced allergic reaction.

**Table 1:** Demographics of physicians surveyed in needs assessment

**Respondent Characteristics**	**# of Respondents**	**% of Respondents**
**Practice Location**		
UC Davis	42	51%
Mount Sinai	40	49%
**Number of Patients with Acute Food Allergy Seen Annually**		
0	16	20%
1–10	54	65%
>10	12	15%
**Number of Patients with History of Food Allergy Seen Annually**		
0	2	2%
1–10	40	49%
>10	40	49%
**Specialty Board Certification**		
Internal Medicine	29	35%
Emergency Medicine	23	28%
Pediatrics	20	24%
Family Practice	11	14%
Other Primary Care (i.e. adolescent health, pediatric emergency medicine, nurse practitioner, physician assistant)	5	6%

Data regarding practice characteristics was collected on each respondent as part of the written survey (n = 82).

In an open-ended question, physicians most frequently (77%) listed self-injectable epinephrine as part of the management plan for an otherwise healthy patient with a documented, life-threatening allergy to peanut or shrimp. The physicians who did not list epinephrine indicated only dietary advice, other treatments, and referral to an allergist as their management plan. On the other hand, all the physicians that noted antihistamine therapy also concurrently listed self-injectable epinephrine. Significantly fewer respondents included each of the other components, particularly education on the signs and symptoms of an allergic reaction or anaphylaxis (Table 2).

**Table 2:** Included components in an emergency food allergy management plan

**Component Included in Emergency Food Allergy Management Plan**	**# of Respondents**	**% of Respondents**
Prescription of epinephrine auto-injector	63	77
Dietary avoidance advice	32	39
Other treatment advice	25	30
Referral to allergist	23	28
Prescription of oral antihistamine	16	19
Recommending medic-alert bracelet	10	12
Prescription of oral corticosteroid	9	11
Referral to online resources/information	2	2
Education on signs and symptoms	2	2
Hospital admission	2	2
Prescription of albuterol metered-dose inhaler	1	1

Respondents selected one or more of listed components they would include in a food allergy management plan (n = 82).

Physicians were then presented with two clinical scenarios involving a tree nut-allergic patient to determine whether their management plan varied based on a history of facial angioedema and either severe or mild wheezing with a prior accidental ingestion. Almost all the respondents (93%) stated that they would advise the patient with a history of severe wheezing to administer self-injectable epinephrine at the onset of a current allergic reaction, whereas 61% of physicians recommended self-injectable epinephrine to the tree-nut allergic patient with a history of mild wheezing.

Two-thirds of respondents reported being comfortable with teaching a patient how to use a self-injectable epinephrine device. However, only 32% had epinephrine trainer devices available at their practice location. Only one-quarter of physicians either provided detailed education on food allergen avoidance or patient education handouts on food allergy. Ninety-eight percent indicated that they had referred a patient with possible life-threatening food allergy to an allergist at some time in their career, and 63% "usually" or "almost always" refer patients. When posed with clinical circumstances, respondents cited the patient experiencing allergic symptoms such as hypotension, urticaria, angioedema, itching, wheezing, or gastrointestinal symptoms with a food exposure as the most common reason for initiating an allergy referral. However, physicians were less likely to refer patients on a limited diet, having a diagnosed food allergy, or having oral pruritus from eating raw fruits or vegetables (Table 3).

**Table 3:** Reasons cited by physicians for initiating an allergy referral

**Indications Cited for Physician Referral to Allergist According to Accepted Guidelines **[15]	**# of Respondents**	**% of Respondents**
Have experienced allergic symptoms (hypotension, urticaria, angioedema, itch, wheezing, gastrointestinal responses) with food exposure	72	88
Limited diet based on perceived adverse reactions to foods	40	49
Have a diagnosed food allergy	28	34
Have experienced itchy mouth from raw fruit/vegetables	23	28

Respondents selected one or more of the listed indications as reason(s) for referral (n = 82).

With regard to prior food allergy training, cumulative clinical experience was the most common modality of education (59% of participants). Written articles (29%), rotations in the emergency department (29%) or allergy clinics (15%), and online (1%) and printed (6%) CME courses were also utilized as sources of information. Twenty percent of participants indicated that they have never received a good presentation on food allergy. On a 4-point Likert scale ranging from "very comfortable" to "uncomfortable", only about half of respondents were "comfortable" to "very comfortable" regarding food allergy diagnosis (54%) and ongoing management of food allergy (48%). Referral guidelines (59%), diagnosis (52%), and patient education (52%) were the areas that physicians desired more information. Small on-site training sessions were selected as the most preferred learning method followed by printed materials such as review articles and self-paced online training modules (Table 4).

**Table 4:** Educational preferences indicated by needs assessment survey

**Education Preferences**	**# of Respondents**	**% of Respondents**
**Topic needing more information**		
Referral guidelines	48	59
Diagnosis	43	52
Providing emergency food allergy action plan	43	52
Educating patients on food allergen avoidance	43	52
Use and indications of self-injectable epinephrine	40	49
Office management of anaphylaxis	33	40
**Learning style preferences**		
Smaller on-site training session	43	52
Printed materials	31	38
Review article in major journal every 2 years	28	34
Self-paced online training modules	22	27
Sessions at large annual professional conference	14	17
Self-paced CD computer training modules	9	11
Self-paced printed training modules	6	7
Other	0	0

Respondents indicated one or more of the listed topics on food allergy as requiring more information and the preferred learning modalities (n = 82).

### Pilot testing of teaching materials

**Figure 1:**
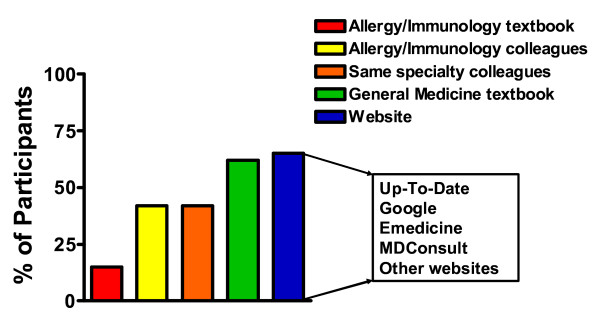
**Types of resources utilized for reference on food allergy**. Pilot program participants indicated whether they have used one or more of the presented resources as a reference on food allergy (n = 26).

Approximately 75 physicians were asked to participate in the pilot testing, and 26 agreed (35% response rate). Participants were certified in one or more of the following: pediatrics (27%), internal medicine (50%), emergency medicine (8%), and other specialties (35%), such as occupational medicine and pediatric emergency medicine. These participants indicated the internet as the most frequently utilized source of information on food allergy, especially websites such as UpToDate, Google, Emedicine, and MD Consult (Figure 1). General medicine or allergy and immunology textbooks, same specialty colleague, or allergy and immunology colleagues were also referred to as information sources.

**Figure 2:**
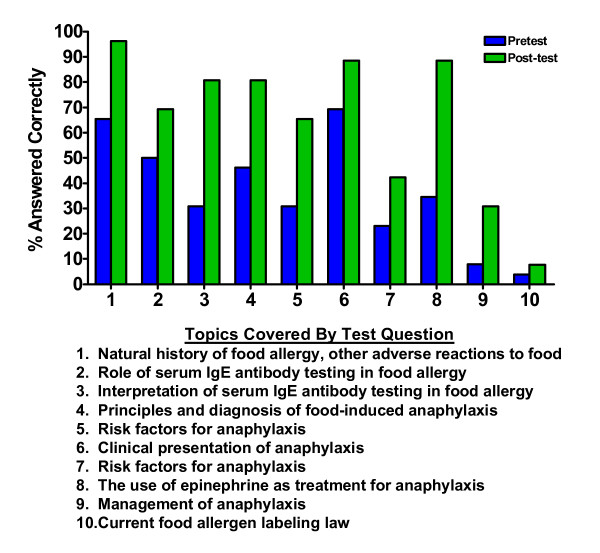
**Item classification and correct response rate by test question**. The % correct response rates are categorized by test question topics (n = 25). One incomplete test was excluded.

There were 25 completed pre- and post-knowledge tests and one incomplete test due to a participant neglecting to answer a question. An overall mean test score of 38% (S.D. = 19%) correct was achieved by participants on the pretest with an increase to 64% (S.D. = 16%) correct on the posttest (p < 0.001). On the pretest, more than half of the participants correctly answered questions pertaining to the natural history of food allergy, other adverse reactions to food, the role of serum IgE antibody testing in food allergy, and the clinical presentation of anaphylaxis (Figure 2). There was an overall improvement on the posttest in the number of correct responses per question with the majority of the healthcare providers correctly answering 7 of the 10 questions. The fewest correct responses on the pre- and post-tests were recorded for the questions specifically pertaining to the risk factors for anaphylaxis, the management of anaphylaxis, and the current food allergen labeling laws.

Most of the participants (81%) indicated that they did not have available epinephrine device trainers at their clinical practice location. Pre-demonstration, 23% of participants were able to demonstrate correct use of the Epipen^® ^device with 85% correctly administering it after the demonstration. One participant recognized the relatively new Twinject^® ^device before the trainer demonstration, but the majority of participants were able to administer the first dose (81%) and the second dose (81%) correctly afterwards at the end of the session.

**Figure 3:**
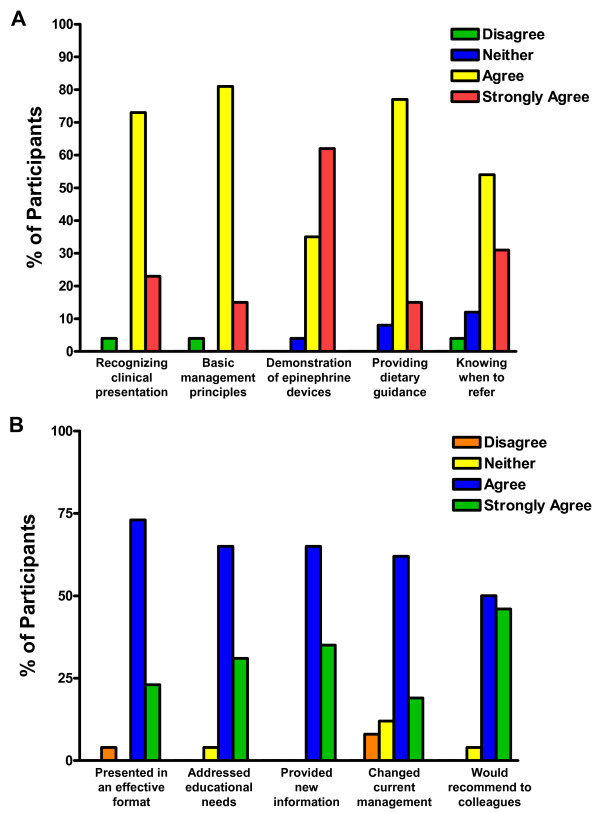
**A) Change in comfort levels in food allergy management after participation in teaching module**. Participants (n = 26) rated on a 5-point Likert scale (from "strongly disagree" to "strongly agree") whether their comfort level improved in performing the listed components of food allergy management.  B) Evaluation of educational aspects of teaching module. Participants (n = 26) rated on a 5-point Likert scale (from "strongly disagree" to "strongly agree") whether the teaching module successfully met the listed educational goals.

The feedback survey revealed that the majority of participants rated "agree" to "strongly agree" that the module increased their comfort with recognition and management of food allergy, providing dietary instructions, and understanding appropriate referral to an allergy specialist (Figure 3A). Most participants indicated that the program was presented in an effective format, included information that was new, met educational needs, and that they would change their anaphylaxis management as a result of the activity (Figure 3B).

## Discussion

Nearly all the primary care and emergency medicine healthcare providers we surveyed treat patients with food allergies. A large number of these healthcare providers received most of their prior training in food allergy through their own personal clinical experience rather than through structured educational programs. Even though many of the participants indicated that they relied on this experience for clinical decision making, most reported being less than comfortable managing food allergy. Physician management plans for life-threatening food allergy were deficient in regards to multiple areas, especially: prescription of self-injectable epinephrine (23% did not include this in a management plan), dietary advice (61% provide none) and referral to an allergist (28% indicated that they "never", "rarely" or only "sometimes" refer). Furthermore, many participants specifically distinguished between mild and severe wheezing as a criterion for prescribing self-injectable epinephrine even though any severity of wheezing in a past reaction is considered a current risk for a severe reaction. Most physicians did not employ educational handouts regarding epinephrine use, and a large number of offices lacked training devices. In addition, there was considerable variation in patient selection and consideration of clinical criteria for initiating an allergy referral, which may pose a barrier for a timely referral to specialist care.

Although many physicians indicated being at least somewhat comfortable with the diagnosis and management of food allergy, there were discrepancies between their reported comfort level and knowledge test scores and self-injectable epinephrine device demonstrations. Nearly two thirds of participants felt comfortable with teaching the epinephrine self-injector technique, but only 23% of the pilot program participants could properly demonstrate Epipen^® ^use before taking the educational module.

We found that there is suboptimal comfort with diagnosis and management of food allergy among primary care and emergency physicians, and many providers indicated a clear interest in further education, particularly regarding referral guidelines, diagnosis, and patient education. Our study showed that most healthcare providers currently utilize many different reference sources for information on the management of food allergies, including other colleagues and medical textbooks. In particular, healthcare providers most frequently turned to the internet for information, which reflects the current trend of increased reliance on the internet as an educational resource for physicians.

Improving healthcare provider education and better delivery of that education via currently employed forms of learning would certainly improve the continuity and quality of care of food allergy patients. However, there are currently limited formal food allergy educational programs designed for and easily accessible by primary care physicians. Our survey results revealed that the preferred methods of education were small conferences, printed materials, and online training. To address these varied learning styles, we selected a PowerPoint slide format as this could be ultimately adapted to either a small conference, individualized instruction, or an online tutorial setting.

Our study demonstrated that this program was beneficial to increasing physician knowledge in food allergy as evidenced by the improvement in test scores and use of self-injectable epinephrine devices. Most participants indicated that the teaching module was presented in an effective format, addressed their educational needs, and increased their knowledge regarding the management of food allergy and anaphylaxis. Also, many respondents (96%) would recommend a similar educational program to their colleagues.

We have presented primarily a descriptive study with several limitations including a small sample size and the recruitment of participants at only two locations at academic centers. Having two study sites could reduce the generalizability of our findings. However, we were successful in surveying physicians of various specialties who practice in a variety of settings. The small size affects the statistical strength of our study, but our data provides compelling observations about food allergy management in primary care. The response rate was generally high among certain venues (staff meetings) while lower when surveys were performed during conferences or sent by interoffice mail. It is difficult to know if physicians with a stronger interest and knowledge in the topic or if physicians with a poor knowledge base seeking additional education were more prone to participate. Nonetheless, overall participation was quite high for the needs assessment (65%) and pilot testing (35%). Additional validation of this program could include administration in other locations and practice environments and to a larger group of physicians and other healthcare providers, such as nurse practitioners and physician assistants. Longitudinal validation is also needed.

Based on the study feedback, we have implemented several modifications in the original slide set and devised a listing of the various relevant websites to accompany the core components of the program. We have also incorporated videos reviewing the use of Epipen^® ^and Twinject^® ^into the PowerPoint module which could be replayed as needed until sufficient mastery of these skills are attained.

## Conclusion

In conclusion, we have developed a flexible educational program that can be an effective teaching tool for primary care and emergency medicine providers for food allergy diagnosis and management. Further testing and validation of this training module will help to maintain its educational value and relevance.

## Competing interests

The authors declare that they have no competing interests.

## Authors' contributions

JEY participated in the design of the study, data acquisition, data analysis and interpretation, and drafting of the manuscript. AK participated in the design of the study, data acquisition, and data analysis and interpretation. CB participated in the design of the study and critical review of the manuscript. SST participated in the study conception, its design and coordination, data acquisition, data analysis and interpretation, and critical review of the manuscript. SHS participated in the study conception, its design and coordination, data analysis and interpretation, and drafting and critical review of the manuscript. All authors read and approved the final manuscript.

## Pre-publication history

The pre-publication history for this paper can be accessed here:



## Supplementary Material

Additional File 1 **Food allergy education teaching module**. This file contains the slides used to pilot the food allergy education program. Videos demonstrating the use of the self-injectable epinephrine devices are embedded in the PowerPoint presentation. The information and revisions in this slide set are the most current as of September 2008. Click here for file
