# Supplementary material for: Development of a food allergy education resource for primary care physicians
Source: BMC Med Educ. 2008 Sep 30;8:45. doi: 10.1186/1472-6920-8-45 (PMC2569928; doi:10.1186/1472-6920-8-45)
Supplement: Additional File 1 — Food allergy education teaching module. This file contains the slides used to pilot the food allergy education program. Videos demonstrating the use of the self-injectable epinephrine devices are embedded in the PowerPoint presentation. The information and revisions in this slide set are the most current as of September 2008. [file 1472-6920-8-45-S1.ppt]

## Slide 1
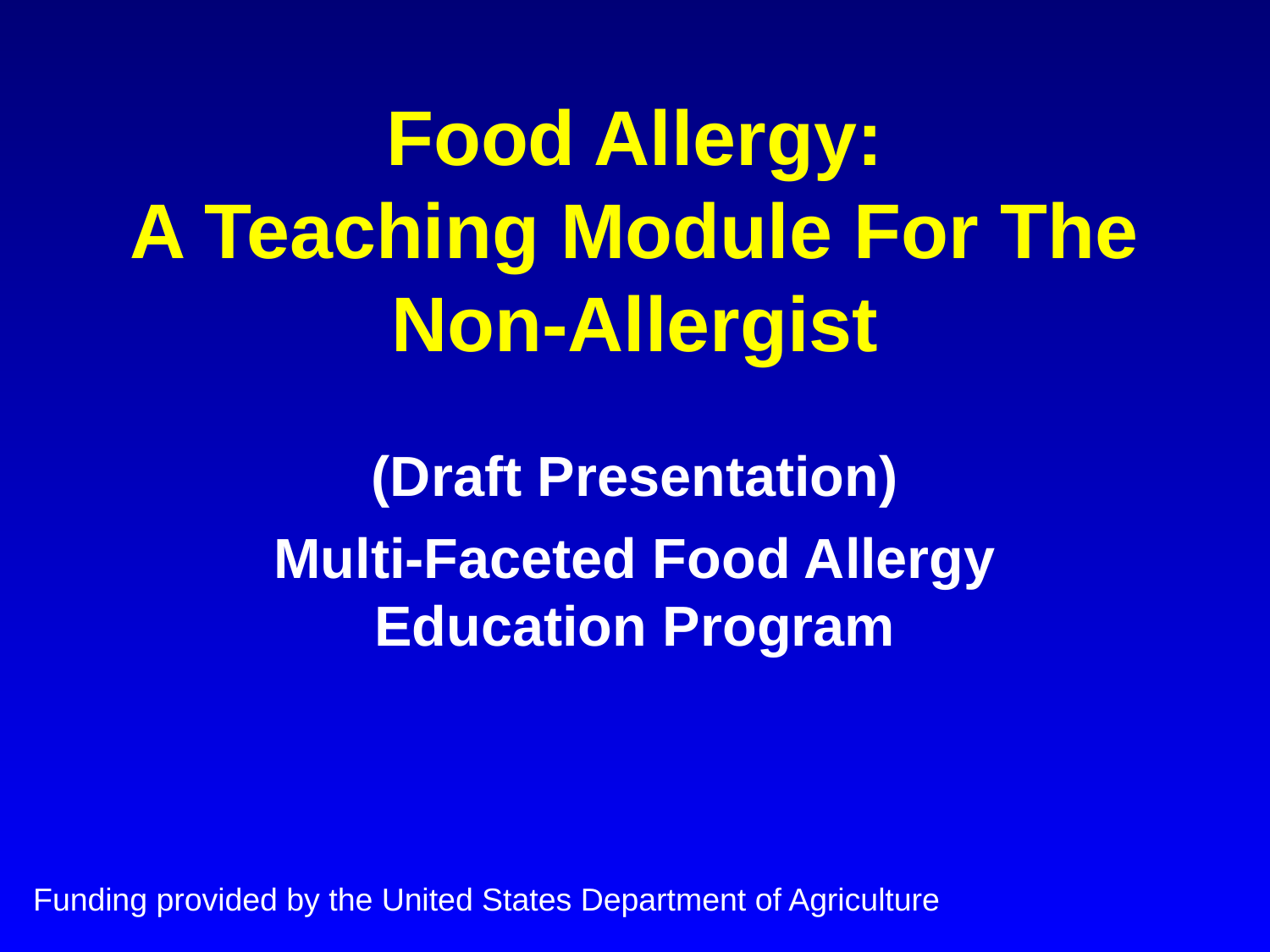

# Food Allergy: A Teaching Module For The Non-Allergist
(Draft Presentation)
Multi-Faceted Food Allergy Education Program
Funding provided by the United States Department of Agriculture

## Slide 2
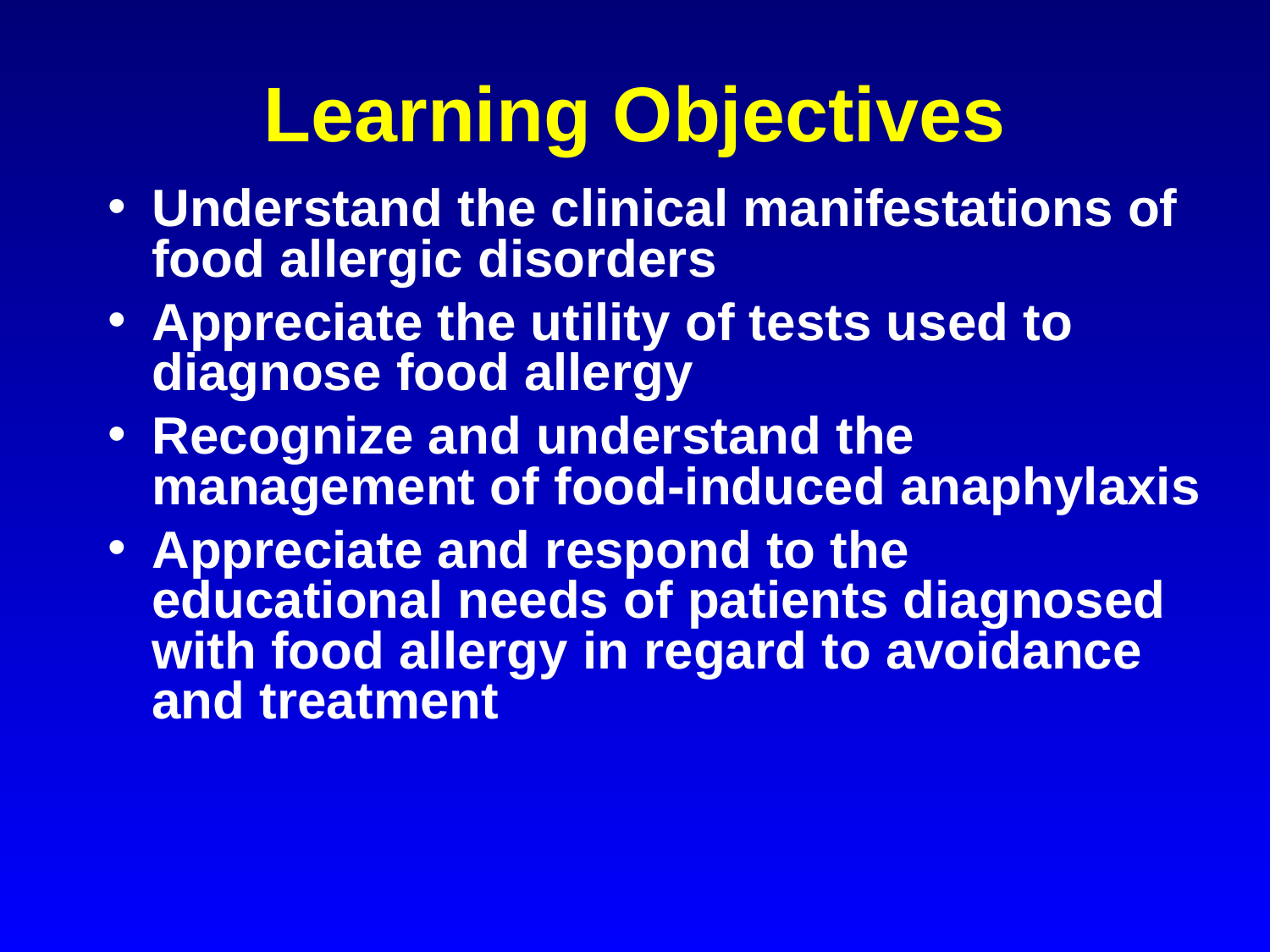

# Learning Objectives
Understand the clinical manifestations of food allergic disorders
Appreciate the utility of tests used to diagnose food allergy
Recognize and understand the management of food-induced anaphylaxis
Appreciate and respond to the educational needs of patients diagnosed with food allergy in regard to avoidance and treatment

## Slide 3
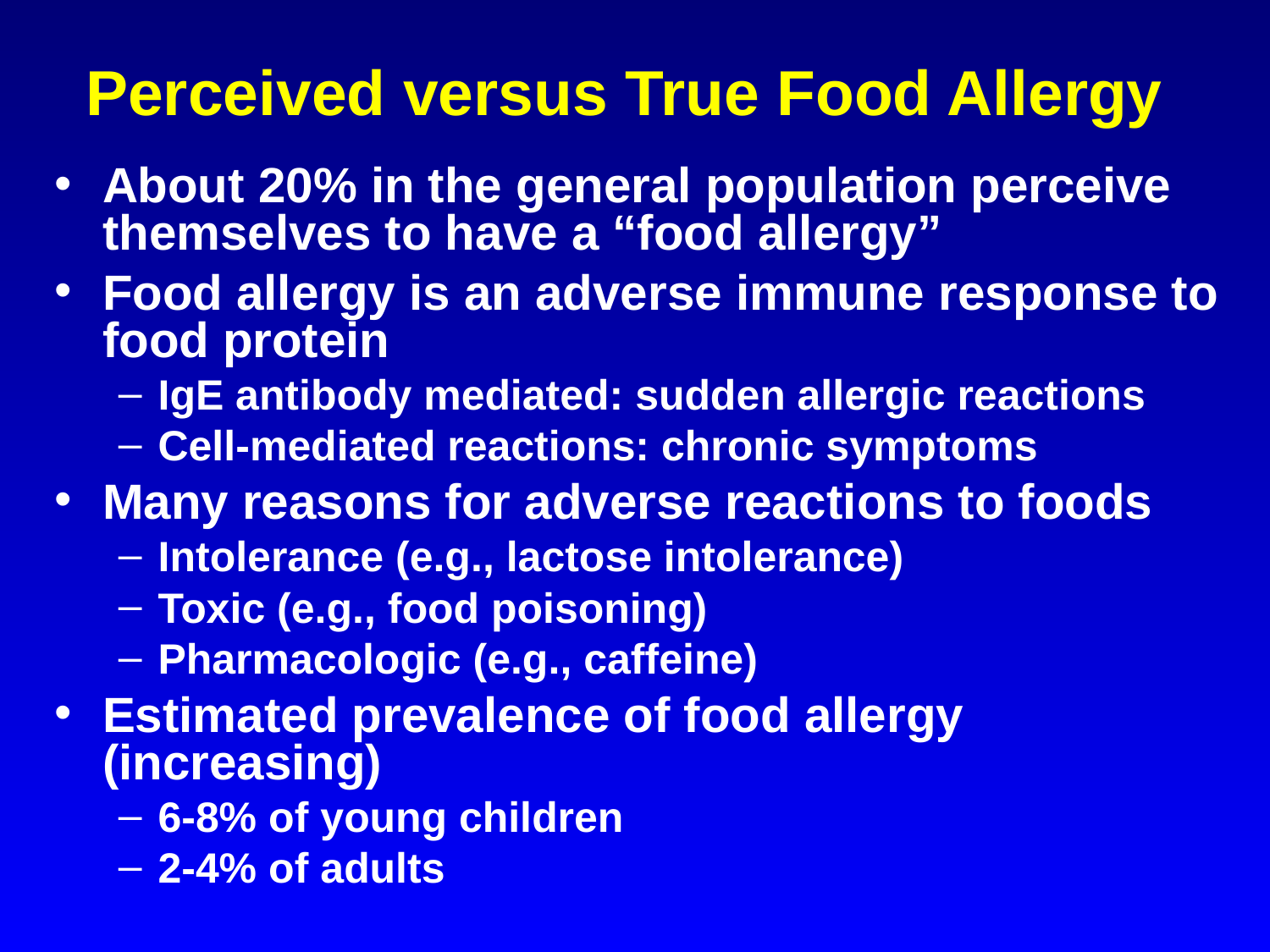

# Perceived versus True Food Allergy
About 20% in the general population perceive themselves to have a “food allergy”
Food allergy is an adverse immune response to food protein
IgE antibody mediated: sudden allergic reactions
Cell-mediated reactions: chronic symptoms
Many reasons for adverse reactions to foods
Intolerance (e.g., lactose intolerance)
Toxic (e.g., food poisoning)
Pharmacologic (e.g., caffeine)
Estimated prevalence of food allergy (increasing)
6-8% of young children
2-4% of adults

## Slide 4
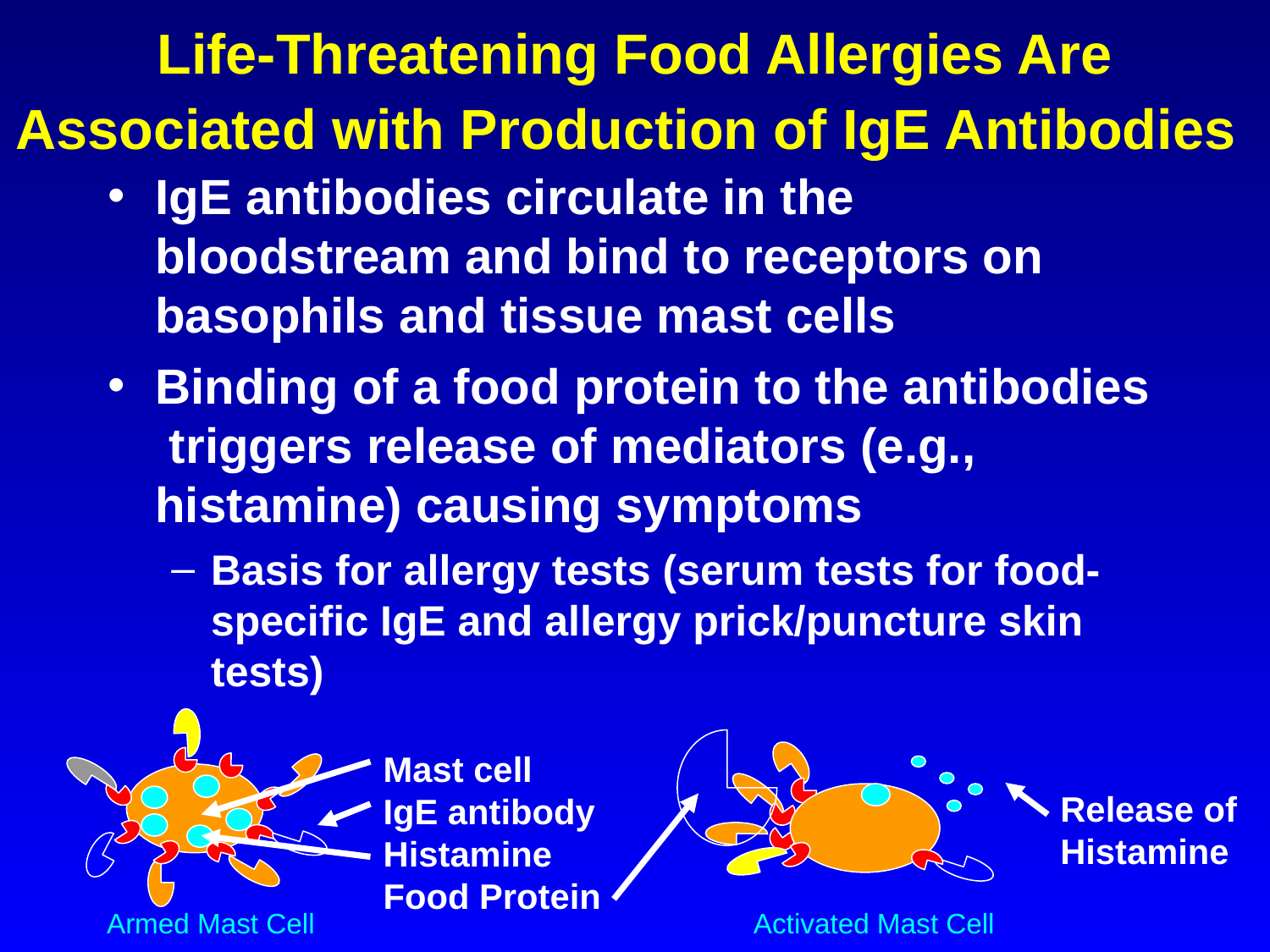

# Life-Threatening Food Allergies Are Associated with Production of IgE Antibodies
IgE antibodies circulate in the bloodstream and bind to receptors on basophils and tissue mast cells
Binding of a food protein to the antibodies triggers release of mediators (e.g., histamine) causing symptoms
Basis for allergy tests (serum tests for food-specific IgE and allergy prick/puncture skin tests)
Mast cell
IgE antibody
Histamine
Food Protein
Release of
Histamine
Armed Mast Cell
Activated Mast Cell

## Slide 5
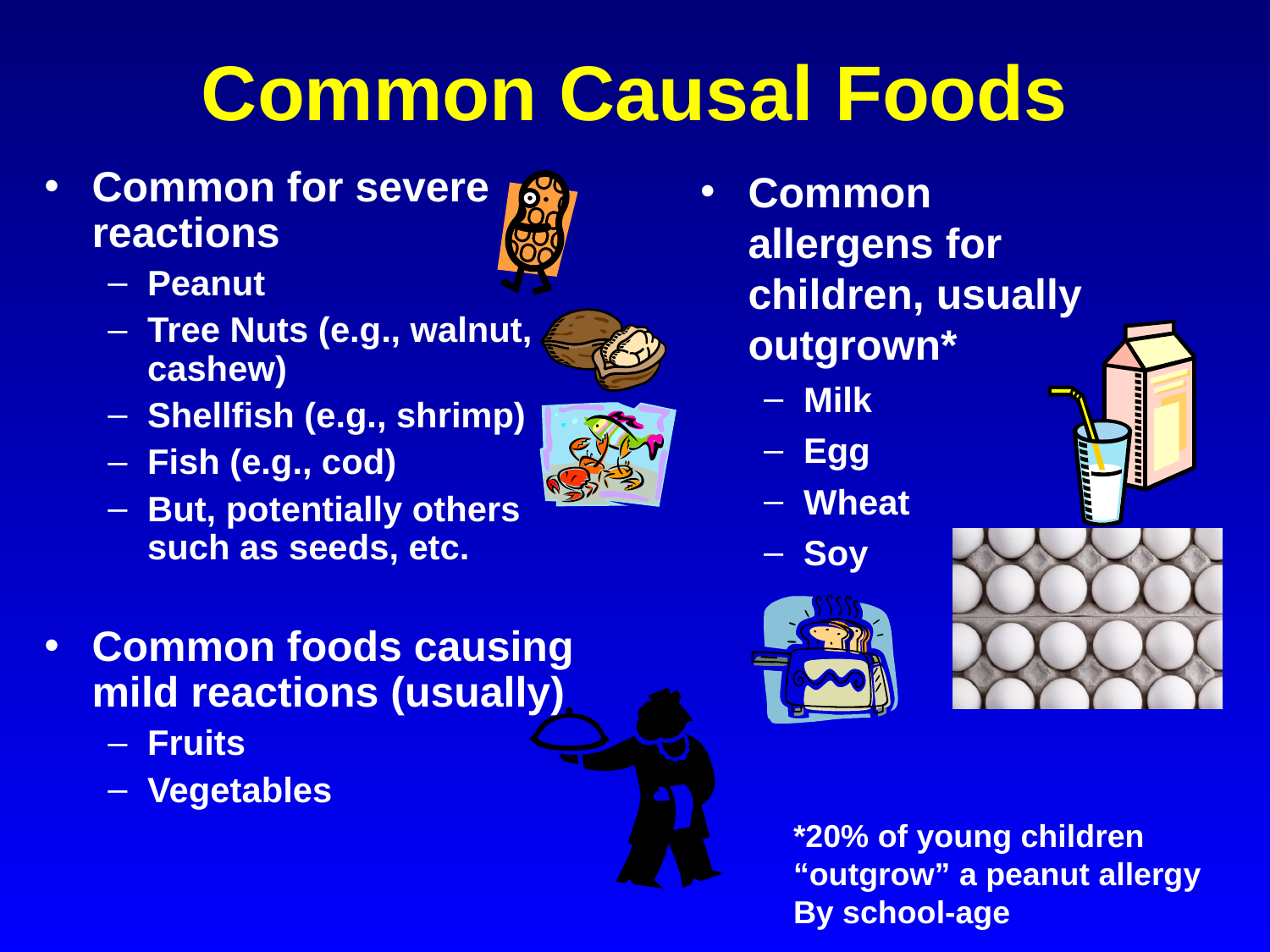

# Common Causal Foods
Common for severe reactions
Peanut
Tree Nuts (e.g., walnut, cashew)
Shellfish (e.g., shrimp)
Fish (e.g., cod)
But, potentially others such as seeds, etc.
Common foods causing mild reactions (usually)
Fruits
Vegetables
Common allergens for children, usually outgrown*
Milk
Egg
Wheat
Soy
*20% of young children
“outgrow” a peanut allergy
By school-age

## Slide 6
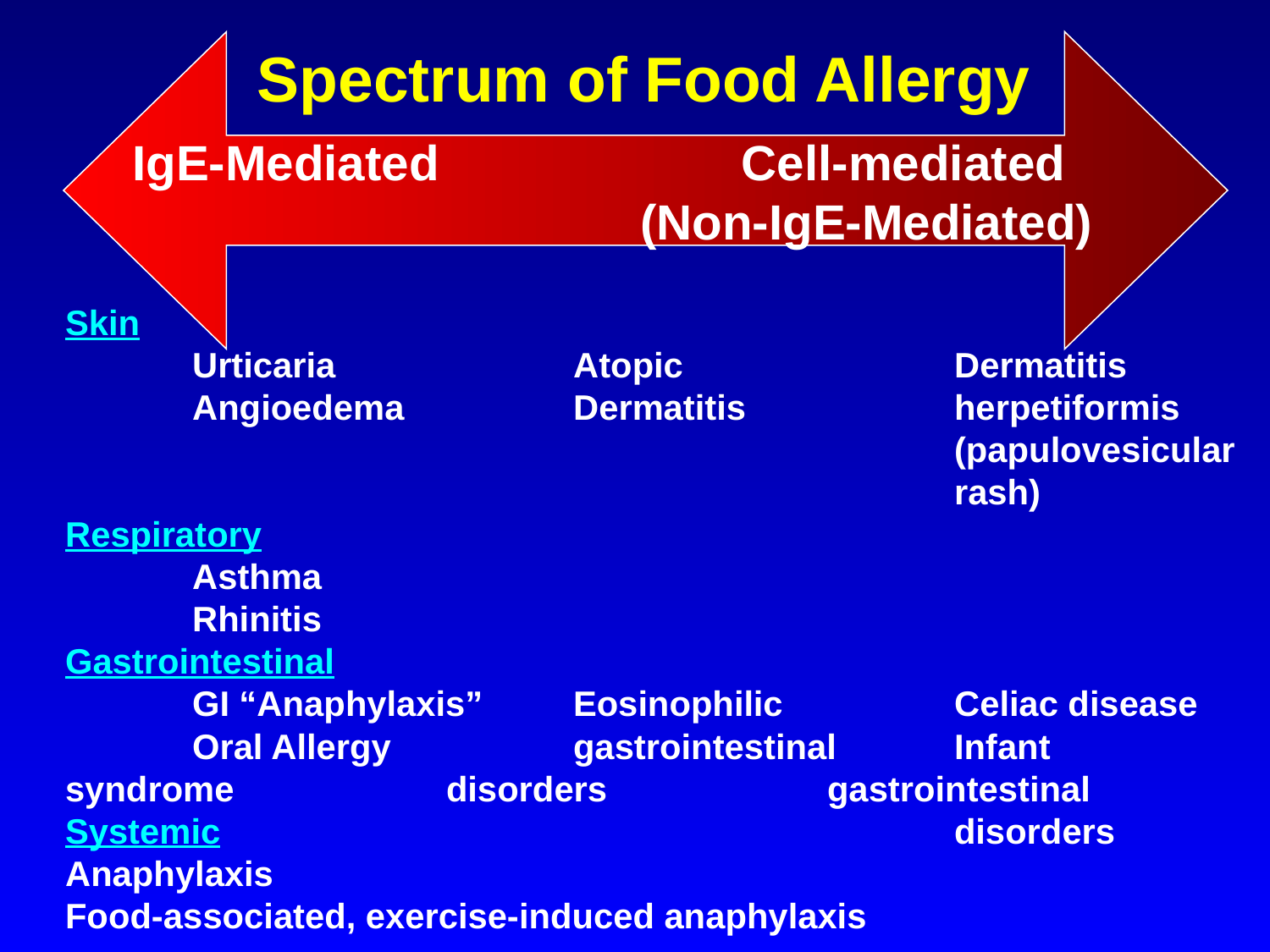

IgE-Mediated Cell-mediated
				(Non-IgE-Mediated)
Spectrum of Food Allergy
Skin
	Urticaria		Atopic 			Dermatitis 		Angioedema 		Dermatitis 		herpetiformis
							(papulovesicular
							rash)
Respiratory
	Asthma
	Rhinitis
Gastrointestinal
	GI “Anaphylaxis”	Eosinophilic 		Celiac disease
	Oral Allergy		gastrointestinal	Infant 		syndrome		disorders		gastrointestinal
Systemic						disorders
Anaphylaxis
Food-associated, exercise-induced anaphylaxis

## Slide 7
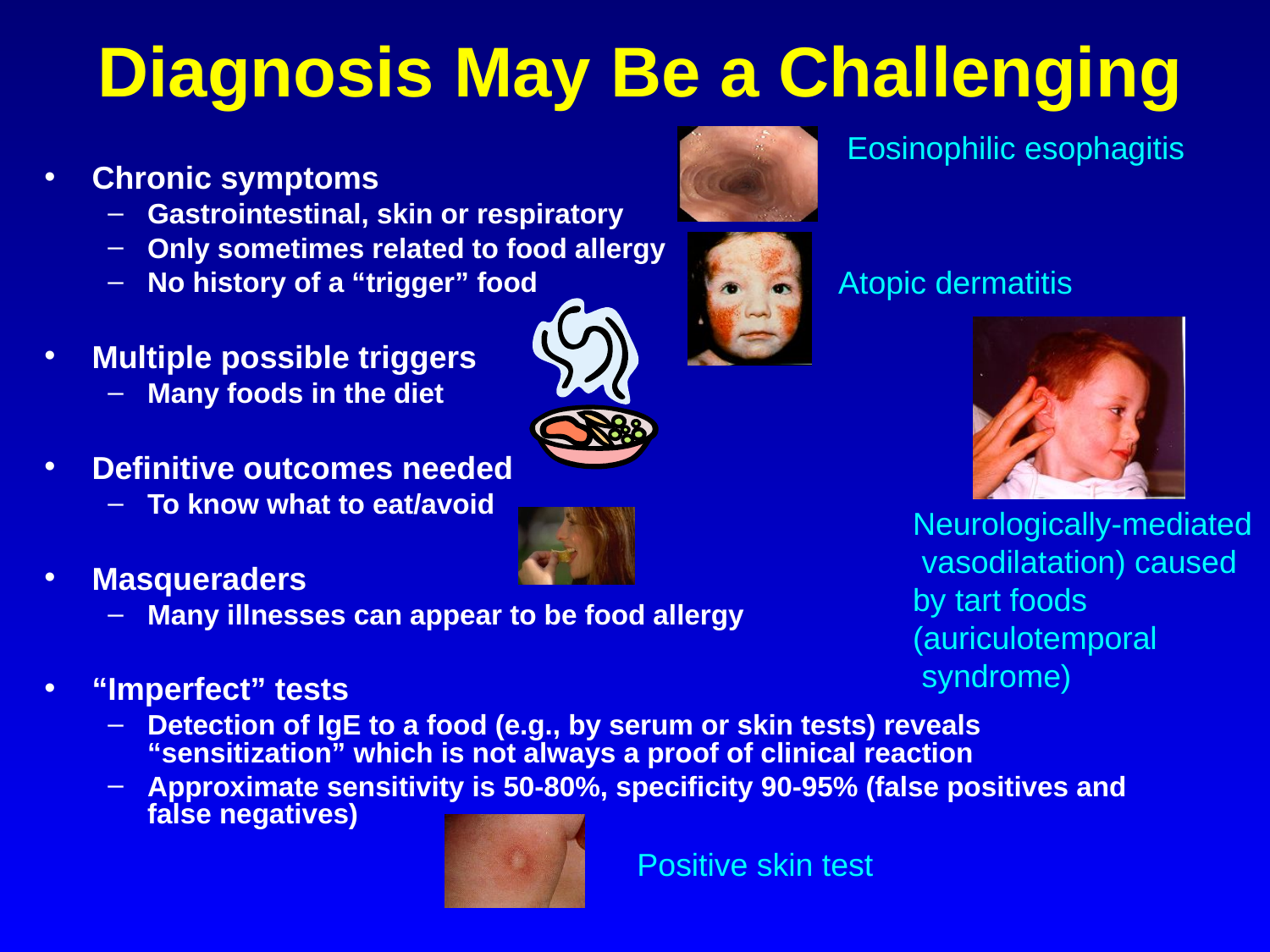

# Diagnosis May Be a Challenging
Eosinophilic esophagitis
Chronic symptoms
Gastrointestinal, skin or respiratory
Only sometimes related to food allergy
No history of a “trigger” food
Multiple possible triggers
Many foods in the diet
Definitive outcomes needed
To know what to eat/avoid
Masqueraders
Many illnesses can appear to be food allergy
“Imperfect” tests
Detection of IgE to a food (e.g., by serum or skin tests) reveals “sensitization” which is not always a proof of clinical reaction
Approximate sensitivity is 50-80%, specificity 90-95% (false positives and false negatives)
Atopic dermatitis
Neurologically-mediated
 vasodilatation) caused
by tart foods
(auriculotemporal
 syndrome)
Positive skin test

## Slide 8
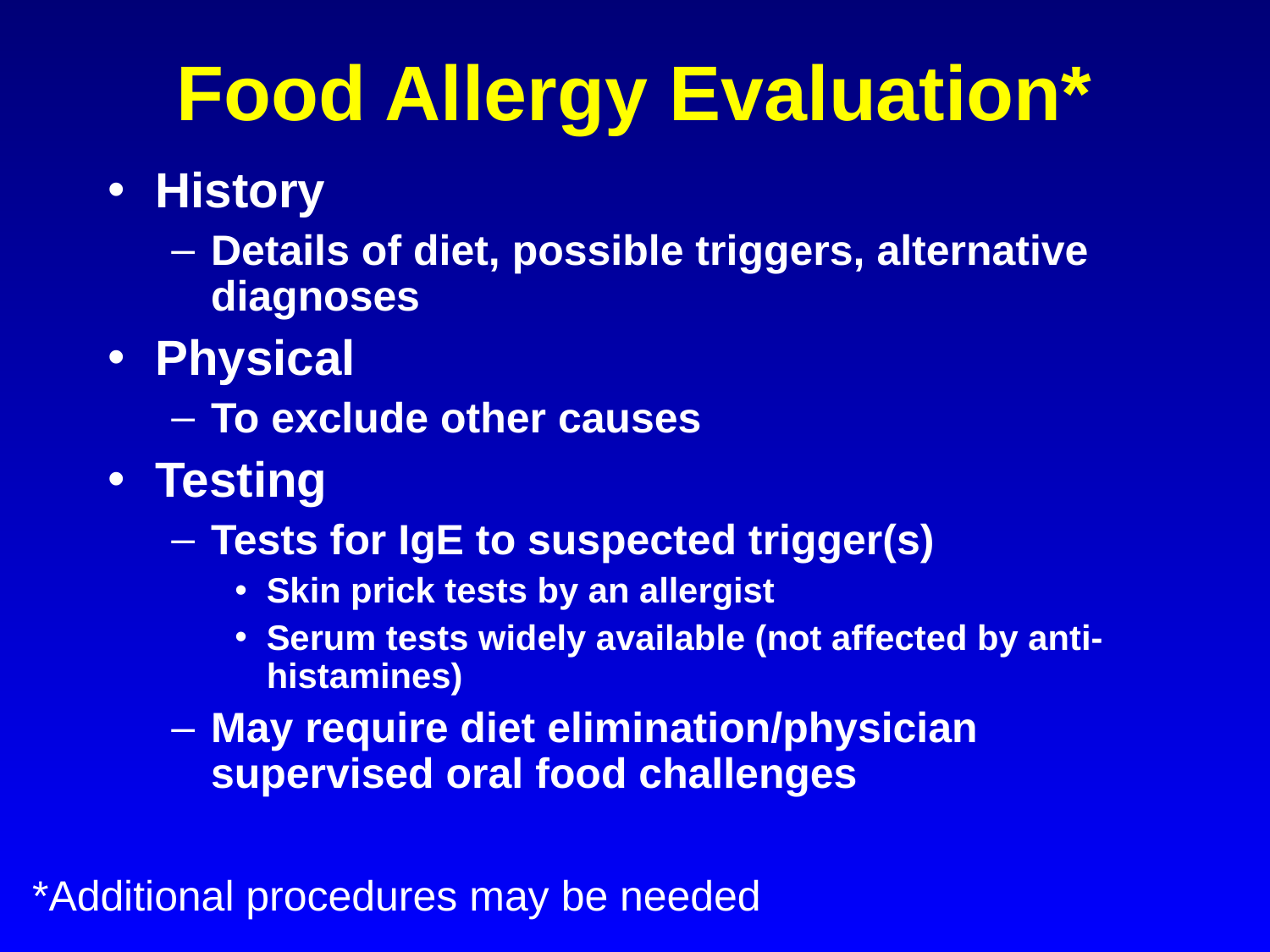

# Food Allergy Evaluation*
History
Details of diet, possible triggers, alternative diagnoses
Physical
To exclude other causes
Testing
Tests for IgE to suspected trigger(s)
Skin prick tests by an allergist
Serum tests widely available (not affected by anti-histamines)
May require diet elimination/physician supervised oral food challenges
*Additional procedures may be needed

## Slide 9
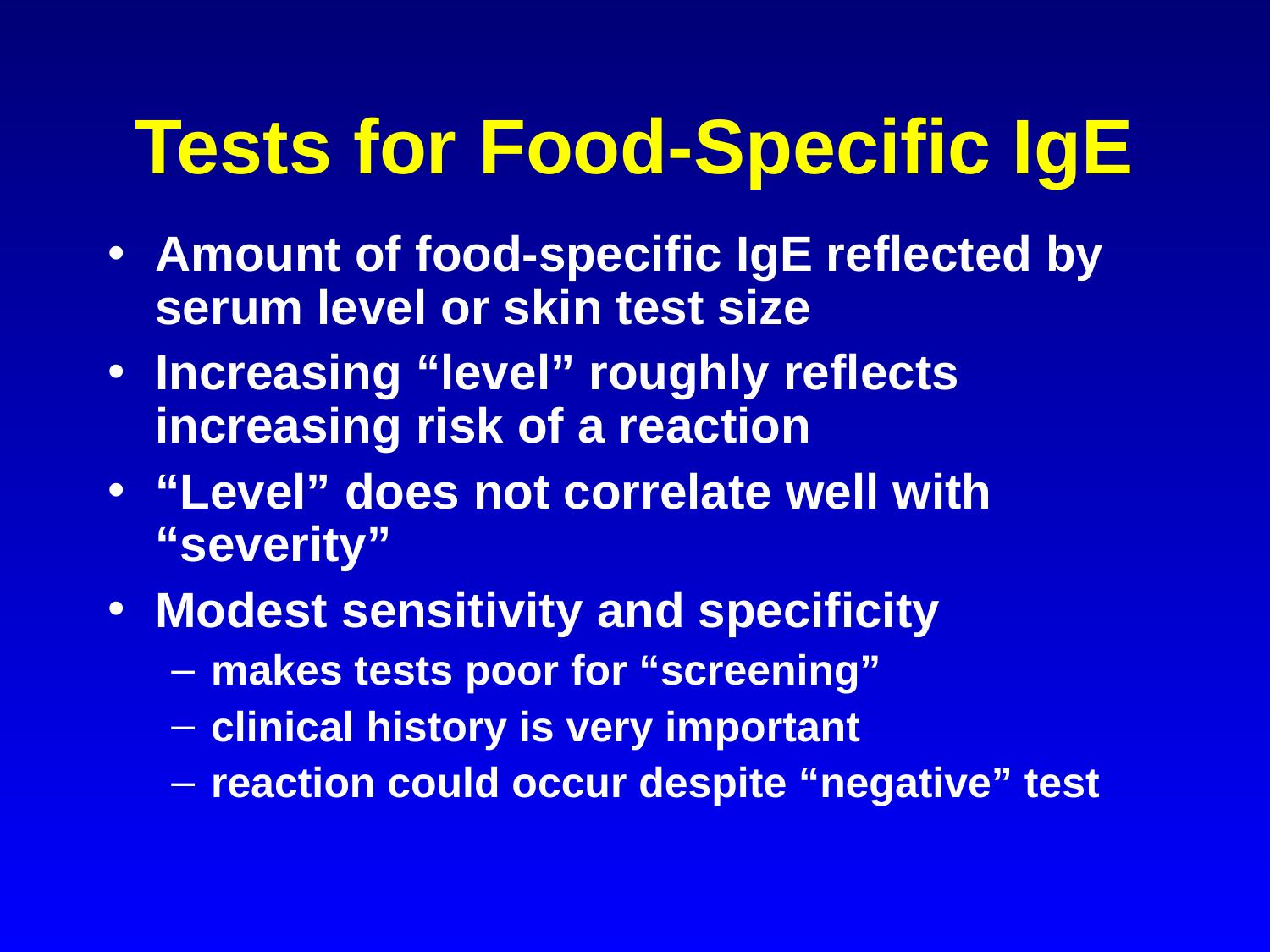

# Tests for Food-Specific IgE
Amount of food-specific IgE reflected by serum level or skin test size
Increasing “level” roughly reflects increasing risk of a reaction
“Level” does not correlate well with “severity”
Modest sensitivity and specificity
makes tests poor for “screening”
clinical history is very important
reaction could occur despite “negative” test

## Slide 10
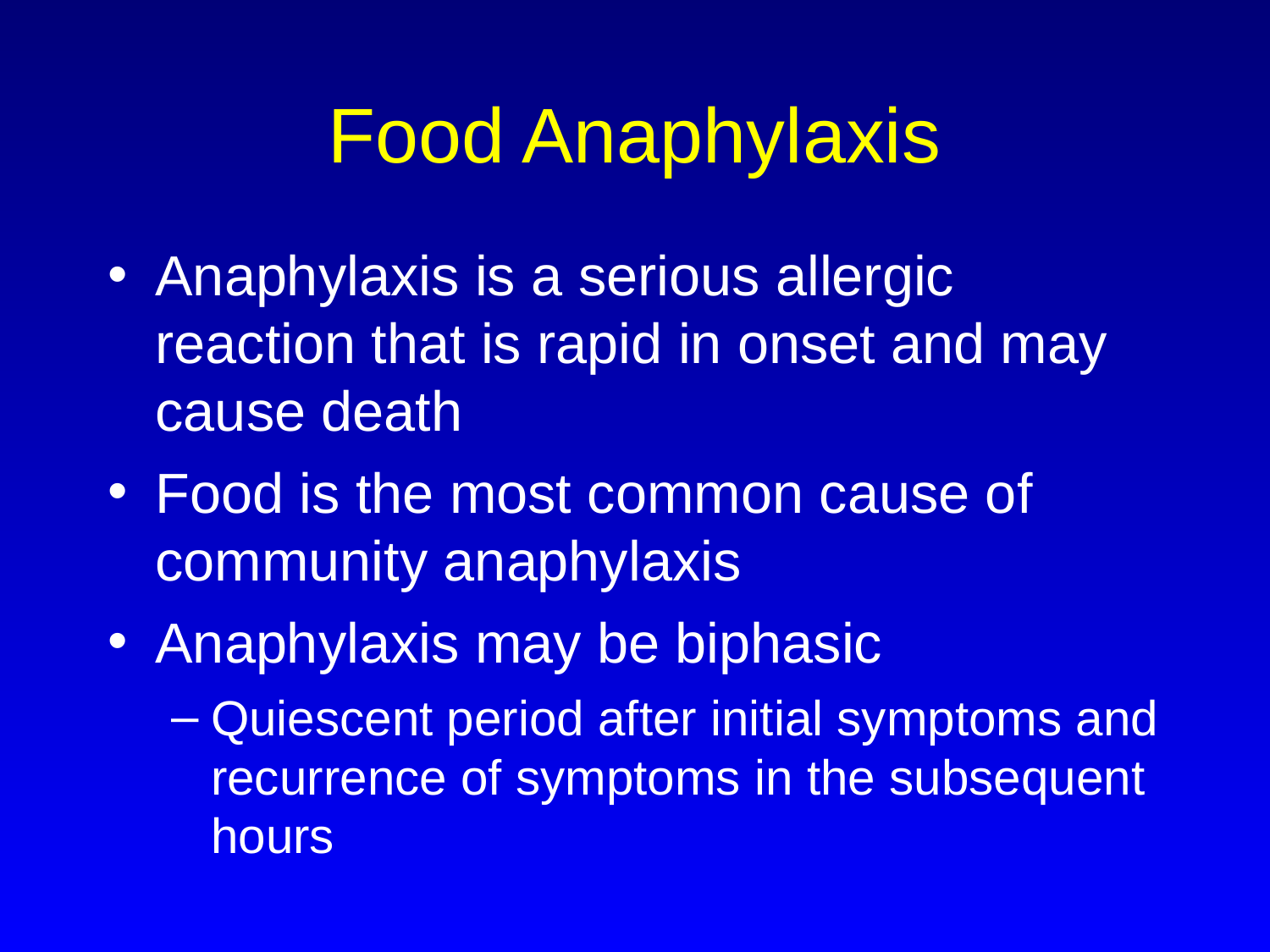

# Food Anaphylaxis
Anaphylaxis is a serious allergic reaction that is rapid in onset and may cause death
Food is the most common cause of community anaphylaxis
Anaphylaxis may be biphasic
Quiescent period after initial symptoms and recurrence of symptoms in the subsequent hours

## Slide 11
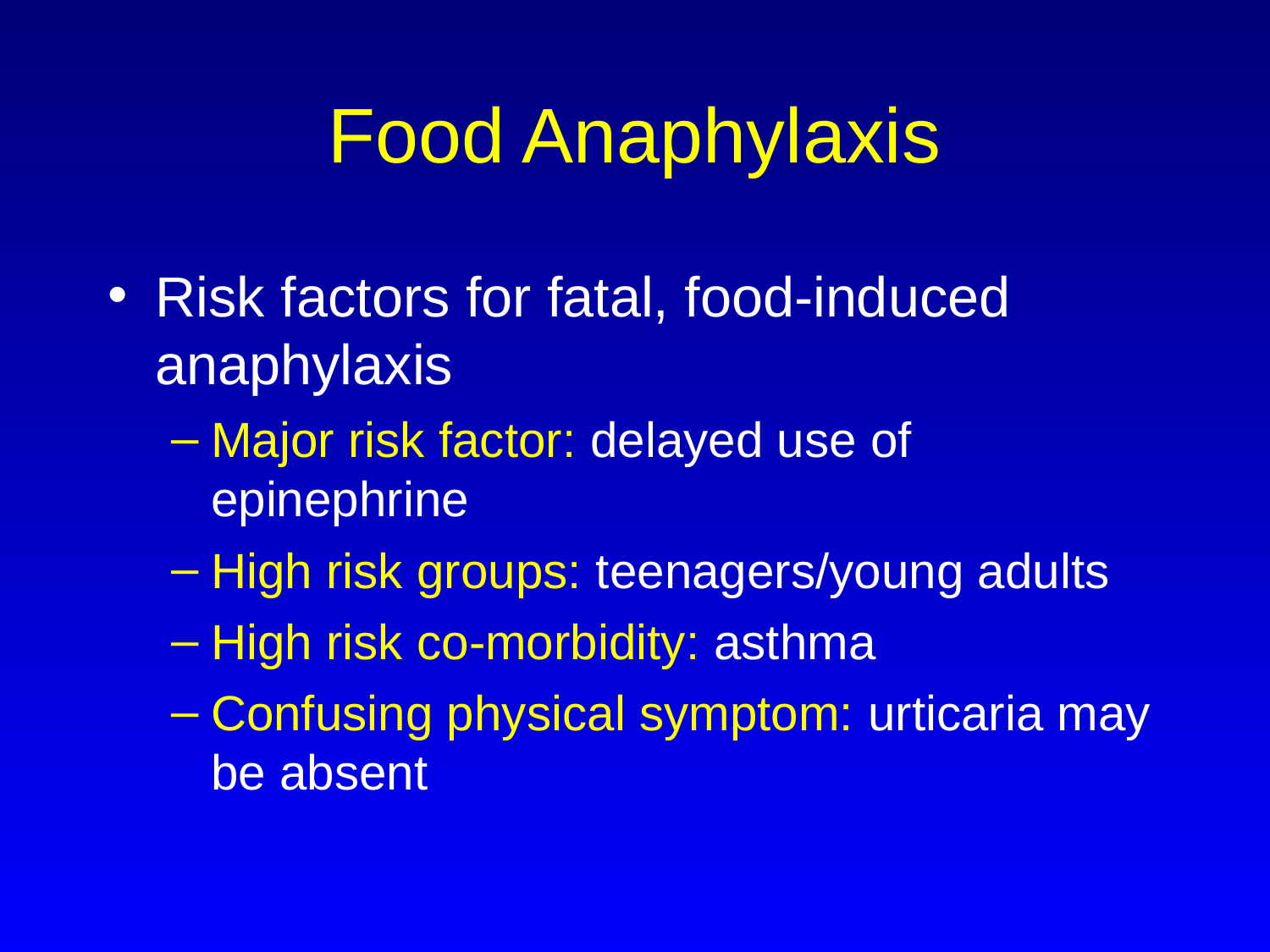

# Food Anaphylaxis
Risk factors for fatal, food-induced anaphylaxis
Major risk factor: delayed use of epinephrine
High risk groups: teenagers/young adults
High risk co-morbidity: asthma
Confusing physical symptom: urticaria may be absent

## Slide 12
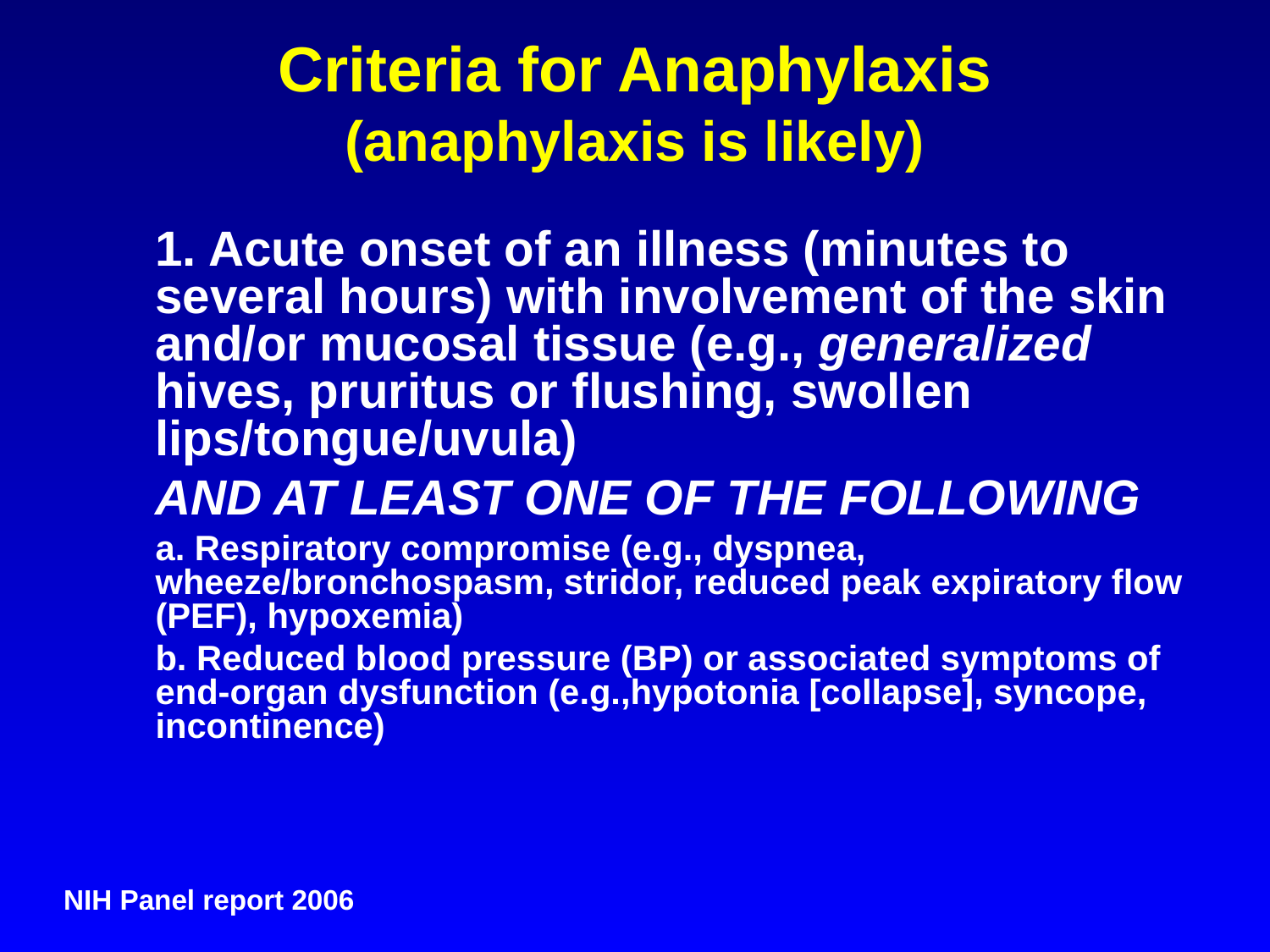

# Criteria for Anaphylaxis (anaphylaxis is likely)
1. Acute onset of an illness (minutes to several hours) with involvement of the skin and/or mucosal tissue (e.g., generalized hives, pruritus or flushing, swollen lips/tongue/uvula)
AND AT LEAST ONE OF THE FOLLOWING
a. Respiratory compromise (e.g., dyspnea, wheeze/bronchospasm, stridor, reduced peak expiratory flow (PEF), hypoxemia)
b. Reduced blood pressure (BP) or associated symptoms of end-organ dysfunction (e.g.,hypotonia [collapse], syncope, incontinence)
NIH Panel report 2006

## Slide 13
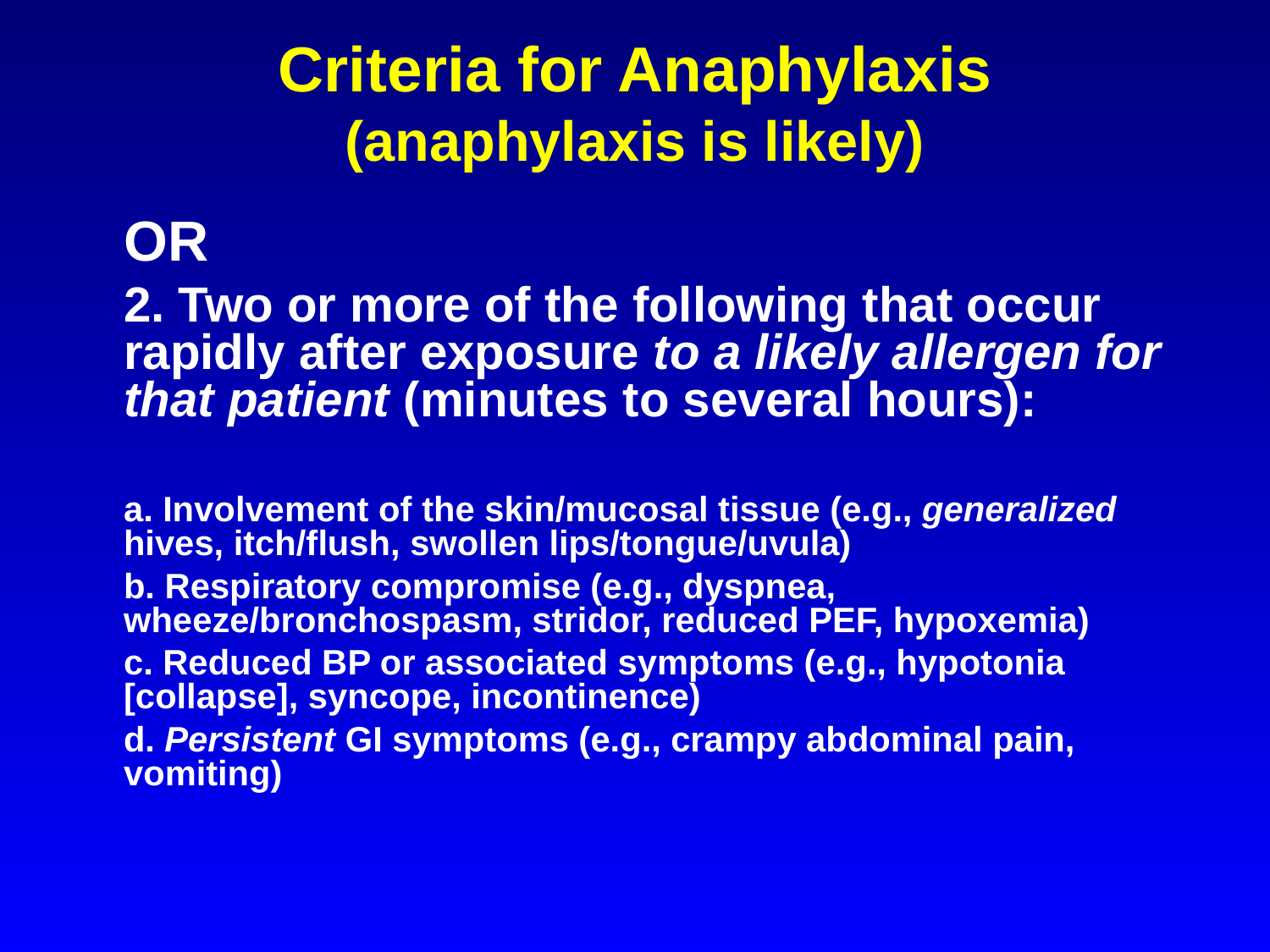

# Criteria for Anaphylaxis (anaphylaxis is likely)
OR
2. Two or more of the following that occur rapidly after exposure to a likely allergen for that patient (minutes to several hours):
a. Involvement of the skin/mucosal tissue (e.g., generalized hives, itch/flush, swollen lips/tongue/uvula)
b. Respiratory compromise (e.g., dyspnea, wheeze/bronchospasm, stridor, reduced PEF, hypoxemia)
c. Reduced BP or associated symptoms (e.g., hypotonia [collapse], syncope, incontinence)
d. Persistent GI symptoms (e.g., crampy abdominal pain, vomiting)

## Slide 14
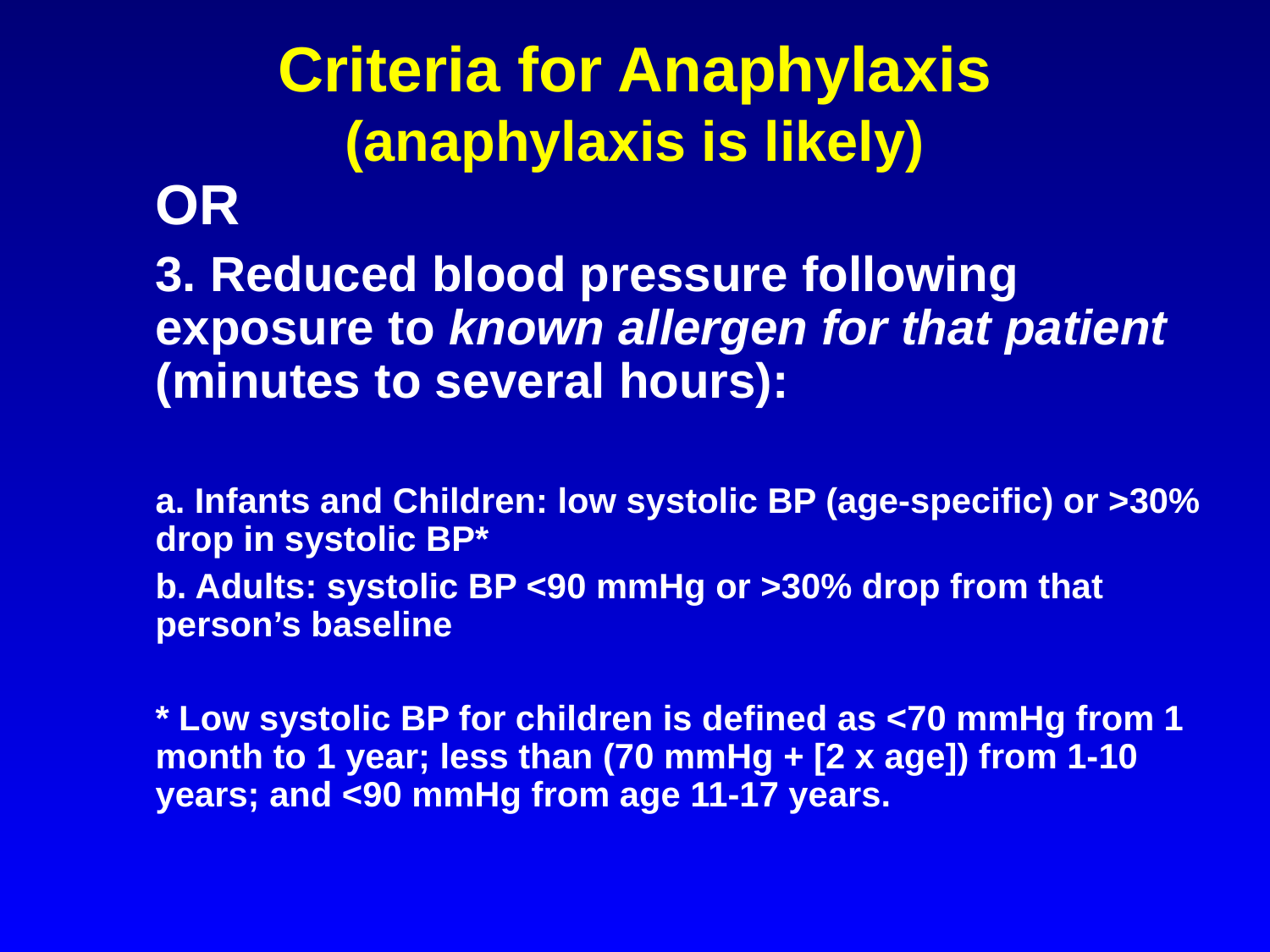

# Criteria for Anaphylaxis (anaphylaxis is likely)
OR
3. Reduced blood pressure following exposure to known allergen for that patient (minutes to several hours):
a. Infants and Children: low systolic BP (age-specific) or >30% drop in systolic BP*
b. Adults: systolic BP <90 mmHg or >30% drop from that person’s baseline
* Low systolic BP for children is defined as <70 mmHg from 1 month to 1 year; less than (70 mmHg + [2 x age]) from 1-10 years; and <90 mmHg from age 11-17 years.

## Slide 15
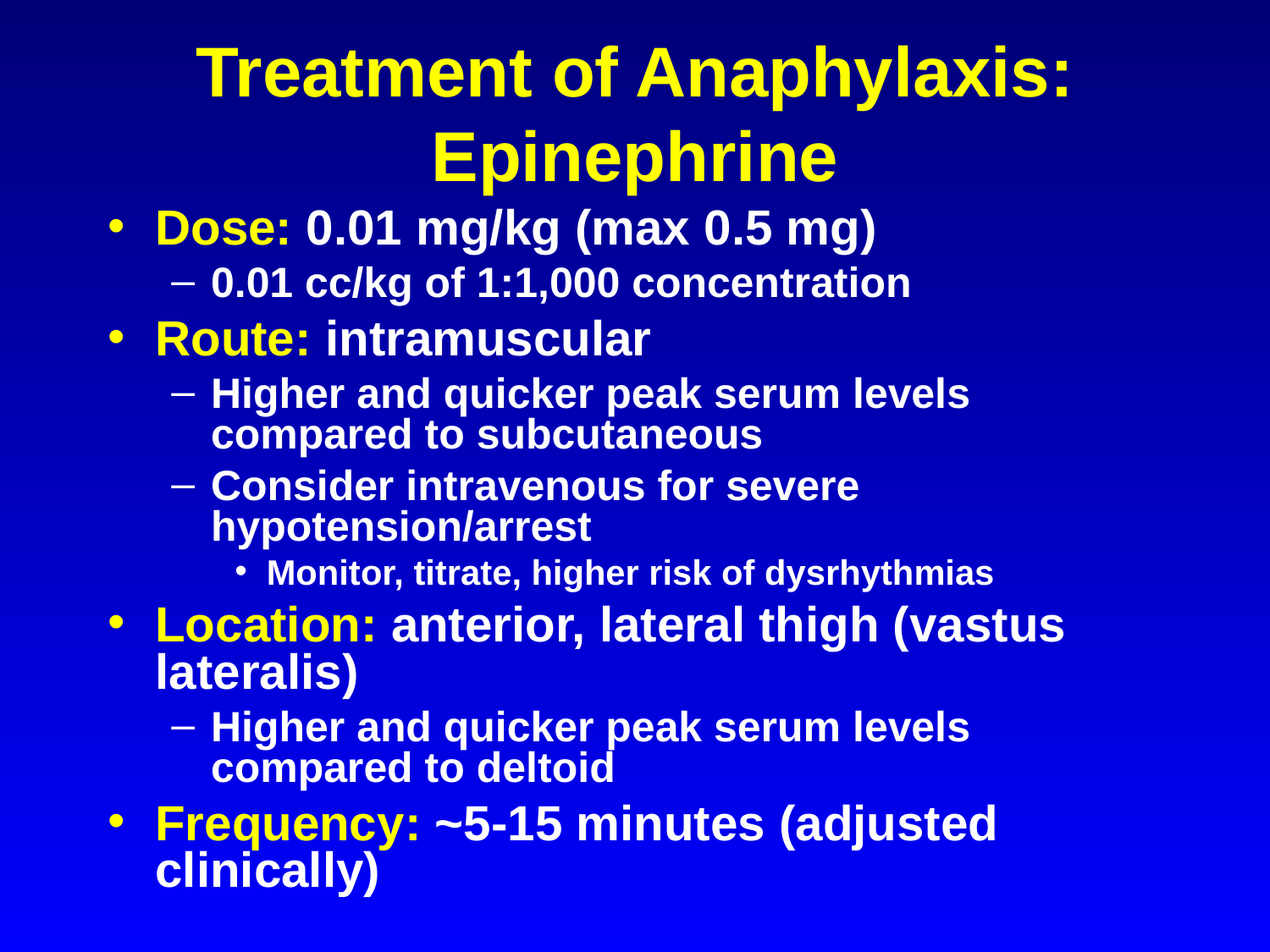

# Treatment of Anaphylaxis: Epinephrine
Dose: 0.01 mg/kg (max 0.5 mg)
0.01 cc/kg of 1:1,000 concentration
Route: intramuscular
Higher and quicker peak serum levels compared to subcutaneous
Consider intravenous for severe hypotension/arrest
Monitor, titrate, higher risk of dysrhythmias
Location: anterior, lateral thigh (vastus lateralis)
Higher and quicker peak serum levels compared to deltoid
Frequency: ~5-15 minutes (adjusted clinically)

## Slide 16
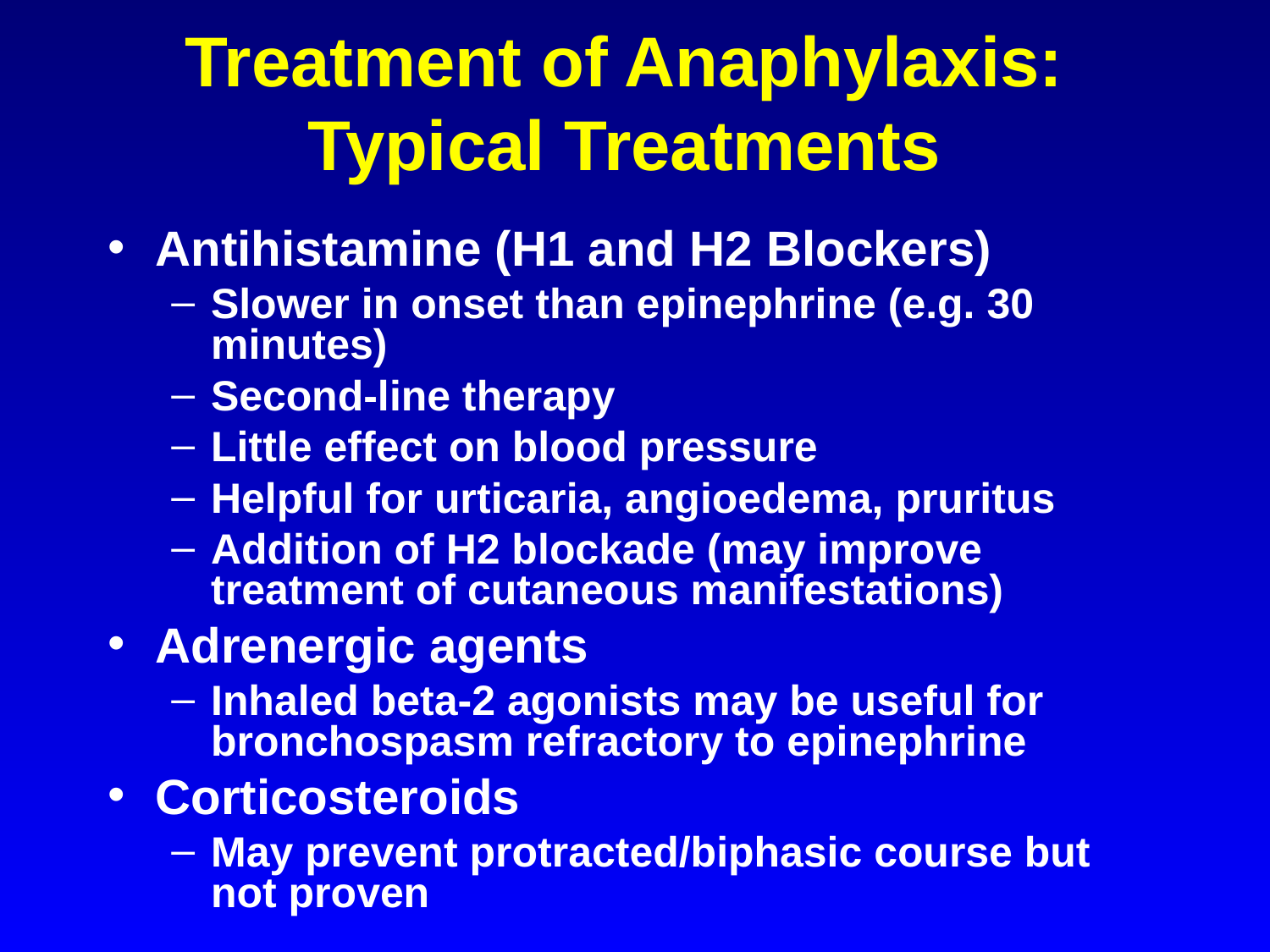

# Treatment of Anaphylaxis: Typical Treatments
Antihistamine (H1 and H2 Blockers)
Slower in onset than epinephrine (e.g. 30 minutes)
Second-line therapy
Little effect on blood pressure
Helpful for urticaria, angioedema, pruritus
Addition of H2 blockade (may improve treatment of cutaneous manifestations)
Adrenergic agents
Inhaled beta-2 agonists may be useful for bronchospasm refractory to epinephrine
Corticosteroids
May prevent protracted/biphasic course but not proven

## Slide 17
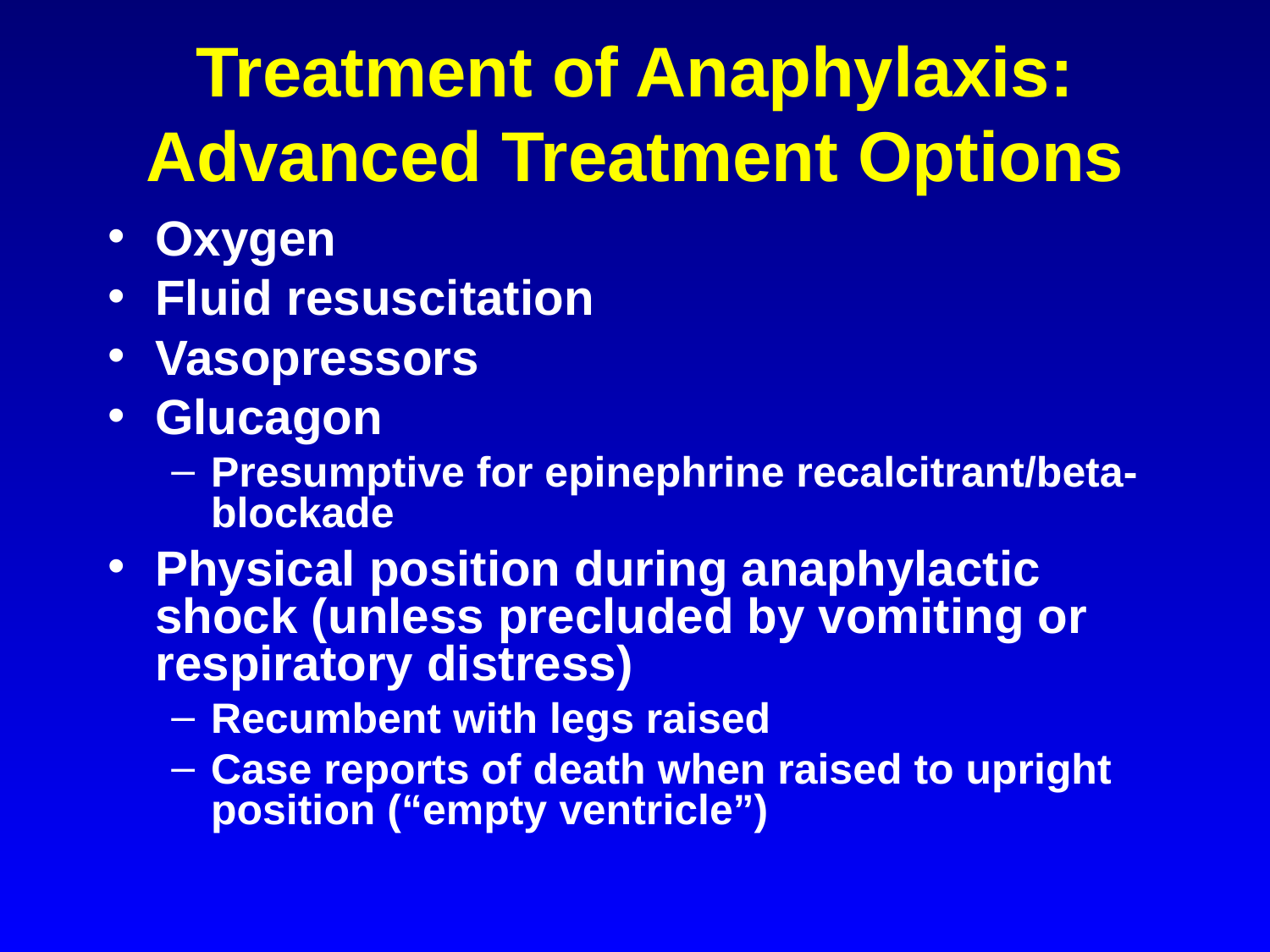

# Treatment of Anaphylaxis: Advanced Treatment Options
Oxygen
Fluid resuscitation
Vasopressors
Glucagon
Presumptive for epinephrine recalcitrant/beta-blockade
Physical position during anaphylactic shock (unless precluded by vomiting or respiratory distress)
Recumbent with legs raised
Case reports of death when raised to upright position (“empty ventricle”)

## Slide 18
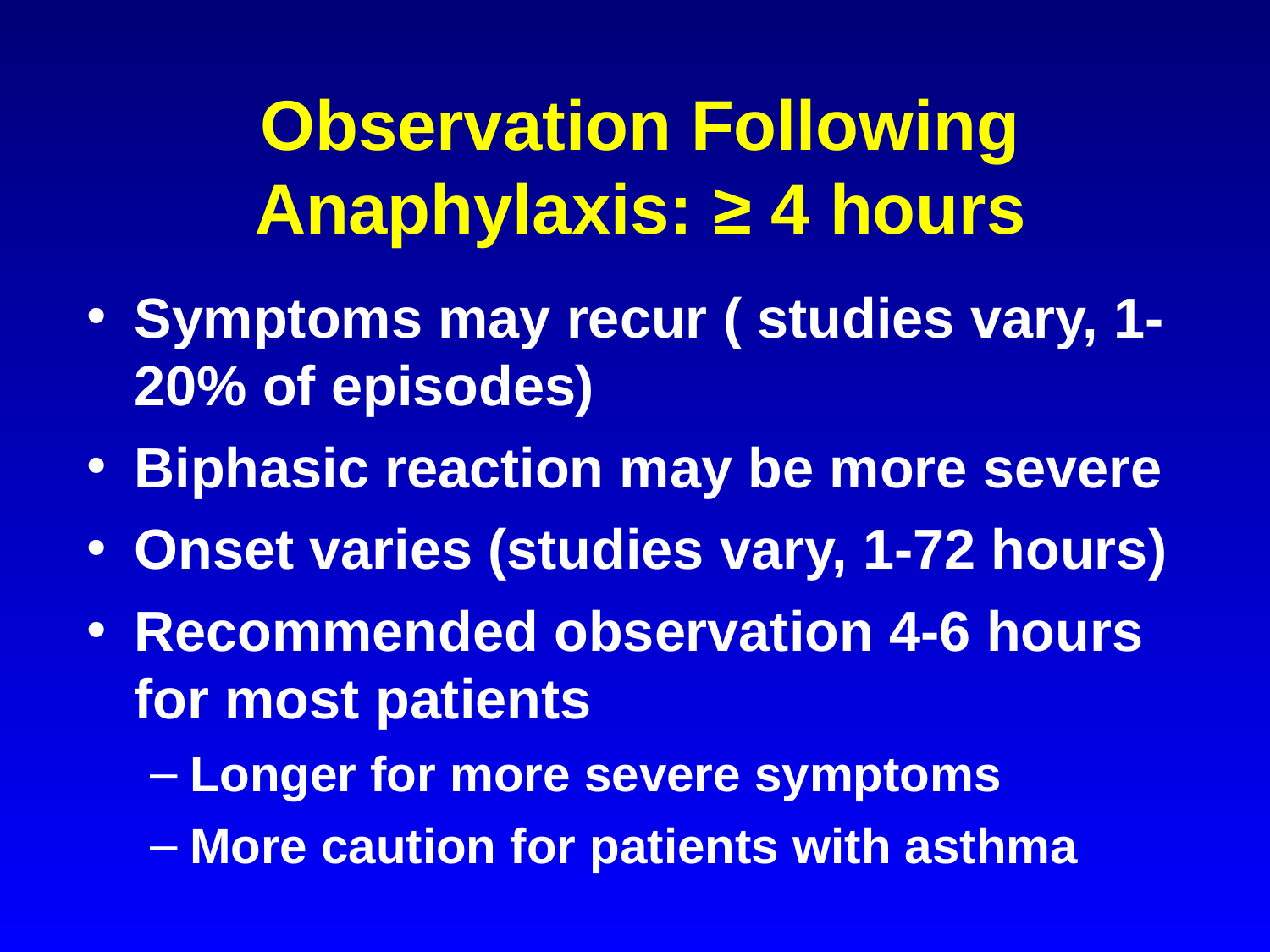

# Observation Following Anaphylaxis: ≥ 4 hours
Symptoms may recur ( studies vary, 1-20% of episodes)
Biphasic reaction may be more severe
Onset varies (studies vary, 1-72 hours)
Recommended observation 4-6 hours for most patients
Longer for more severe symptoms
More caution for patients with asthma

## Slide 19
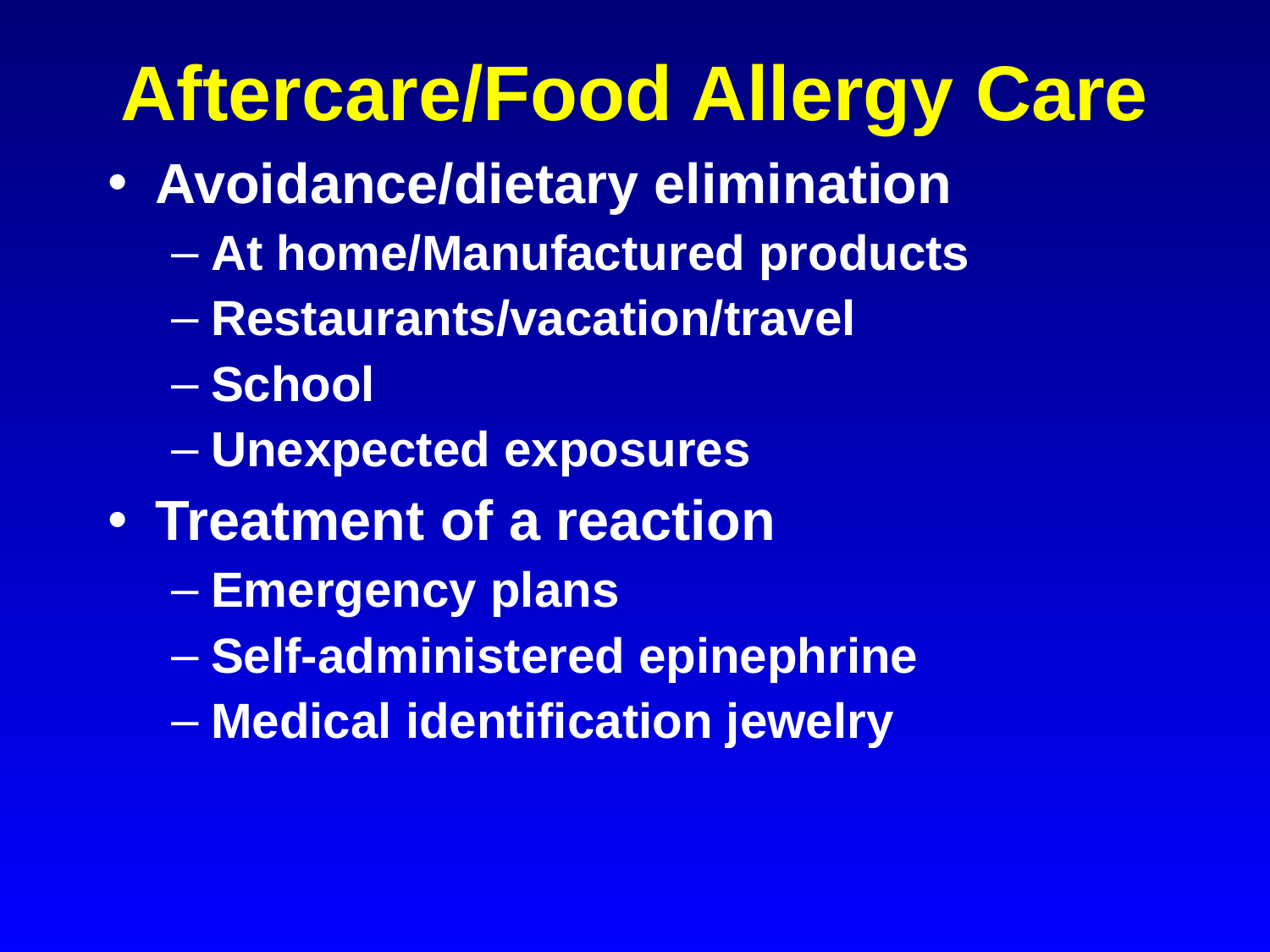

# Aftercare/Food Allergy Care
Avoidance/dietary elimination
At home/Manufactured products
Restaurants/vacation/travel
School
Unexpected exposures
Treatment of a reaction
Emergency plans
Self-administered epinephrine
Medical identification jewelry

## Slide 20
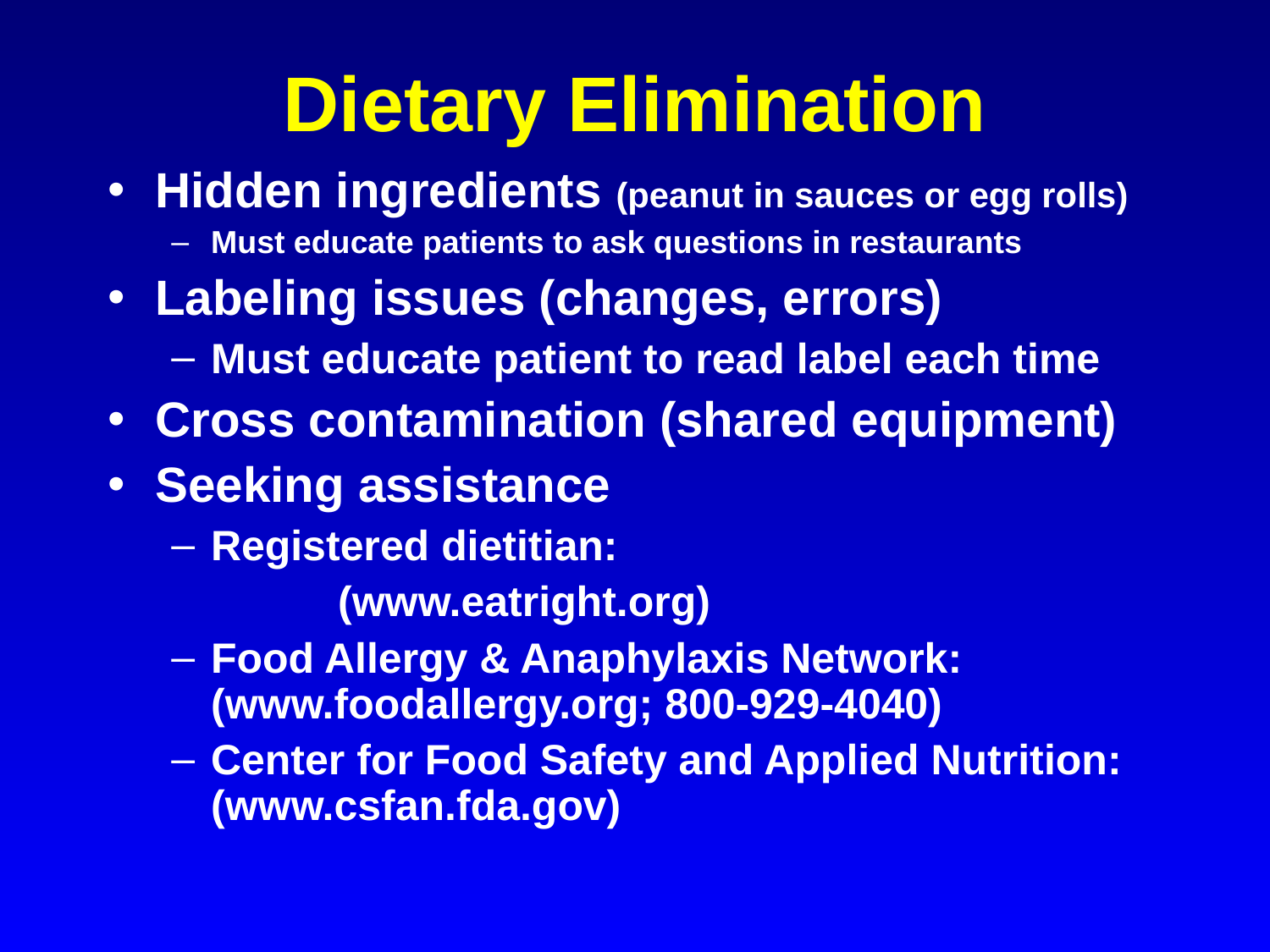

# Dietary Elimination
Hidden ingredients (peanut in sauces or egg rolls)
Must educate patients to ask questions in restaurants
Labeling issues (changes, errors)
Must educate patient to read label each time
Cross contamination (shared equipment)
Seeking assistance
Registered dietitian:
	(www.eatright.org)
Food Allergy & Anaphylaxis Network: (www.foodallergy.org; 800-929-4040)
Center for Food Safety and Applied Nutrition: (www.csfan.fda.gov)

## Slide 21
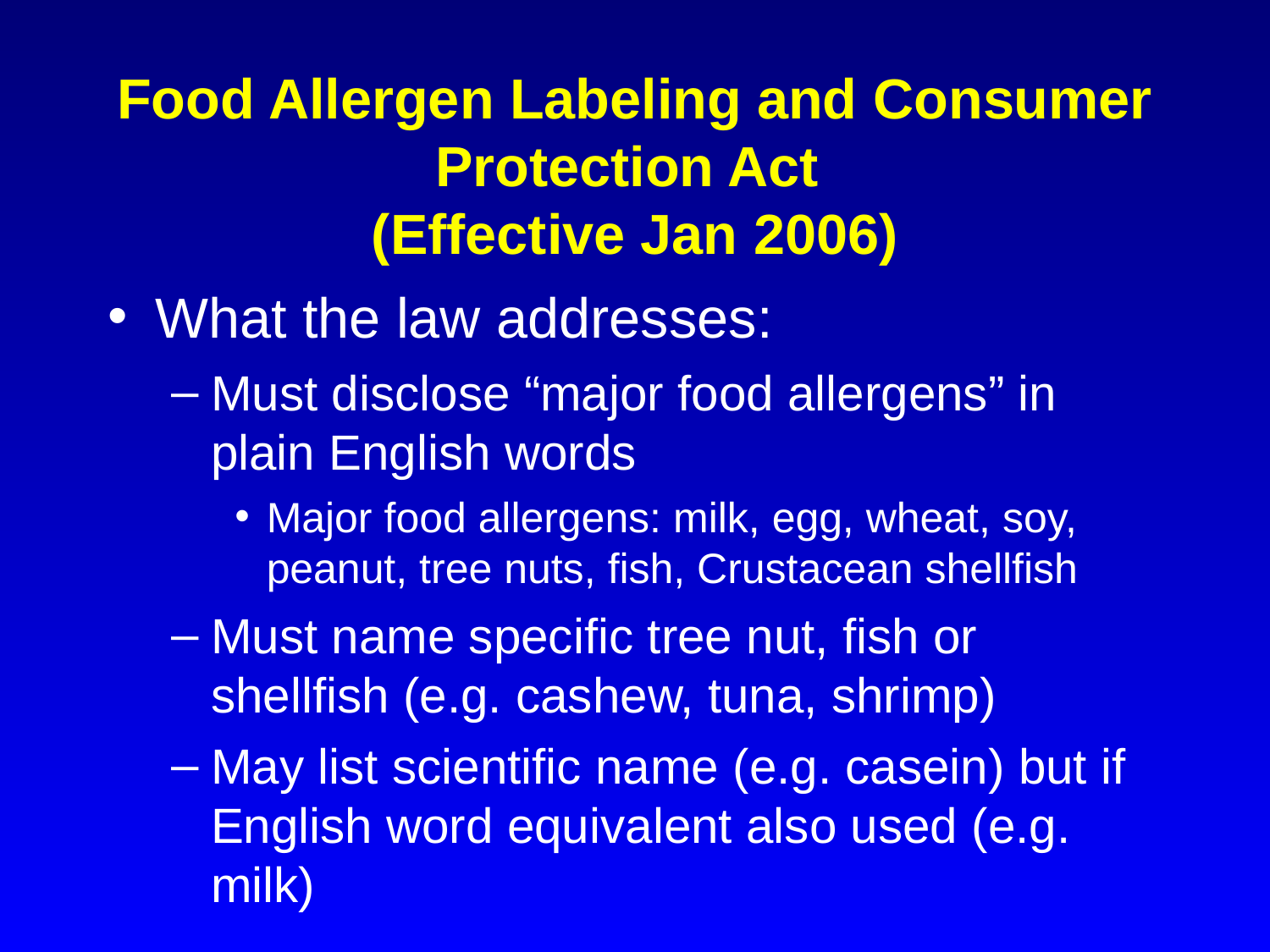

# Food Allergen Labeling and Consumer Protection Act (Effective Jan 2006)
What the law addresses:
Must disclose “major food allergens” in plain English words
Major food allergens: milk, egg, wheat, soy, peanut, tree nuts, fish, Crustacean shellfish
Must name specific tree nut, fish or shellfish (e.g. cashew, tuna, shrimp)
May list scientific name (e.g. casein) but if English word equivalent also used (e.g. milk)

## Slide 22
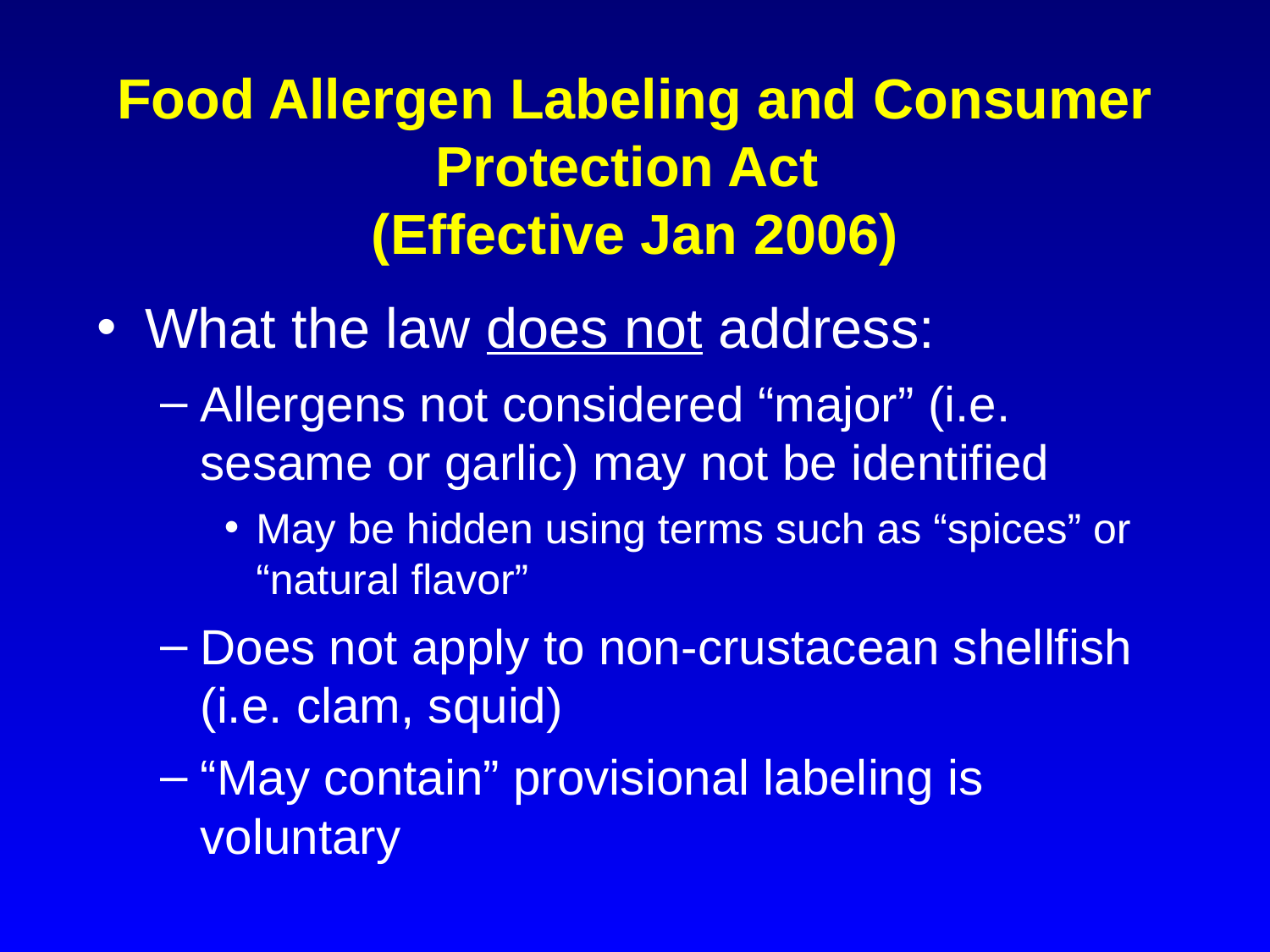

# Food Allergen Labeling and Consumer Protection Act (Effective Jan 2006)
What the law does not address:
Allergens not considered “major” (i.e. sesame or garlic) may not be identified
May be hidden using terms such as “spices” or “natural flavor”
Does not apply to non-crustacean shellfish (i.e. clam, squid)
“May contain” provisional labeling is voluntary

## Slide 23
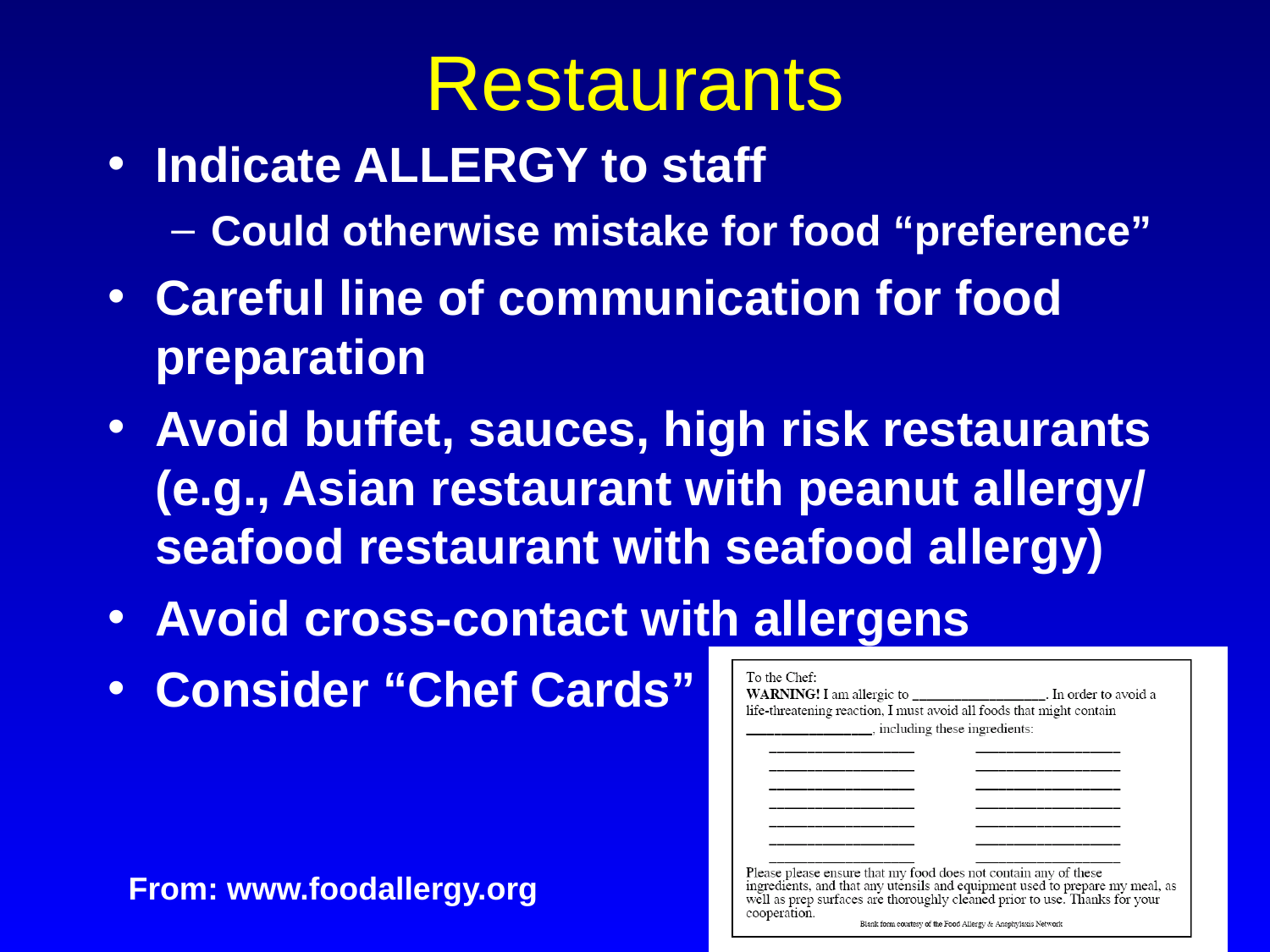

# Restaurants
Indicate ALLERGY to staff
Could otherwise mistake for food “preference”
Careful line of communication for food preparation
Avoid buffet, sauces, high risk restaurants (e.g., Asian restaurant with peanut allergy/ seafood restaurant with seafood allergy)
Avoid cross-contact with allergens
Consider “Chef Cards”
From: www.foodallergy.org

## Slide 24
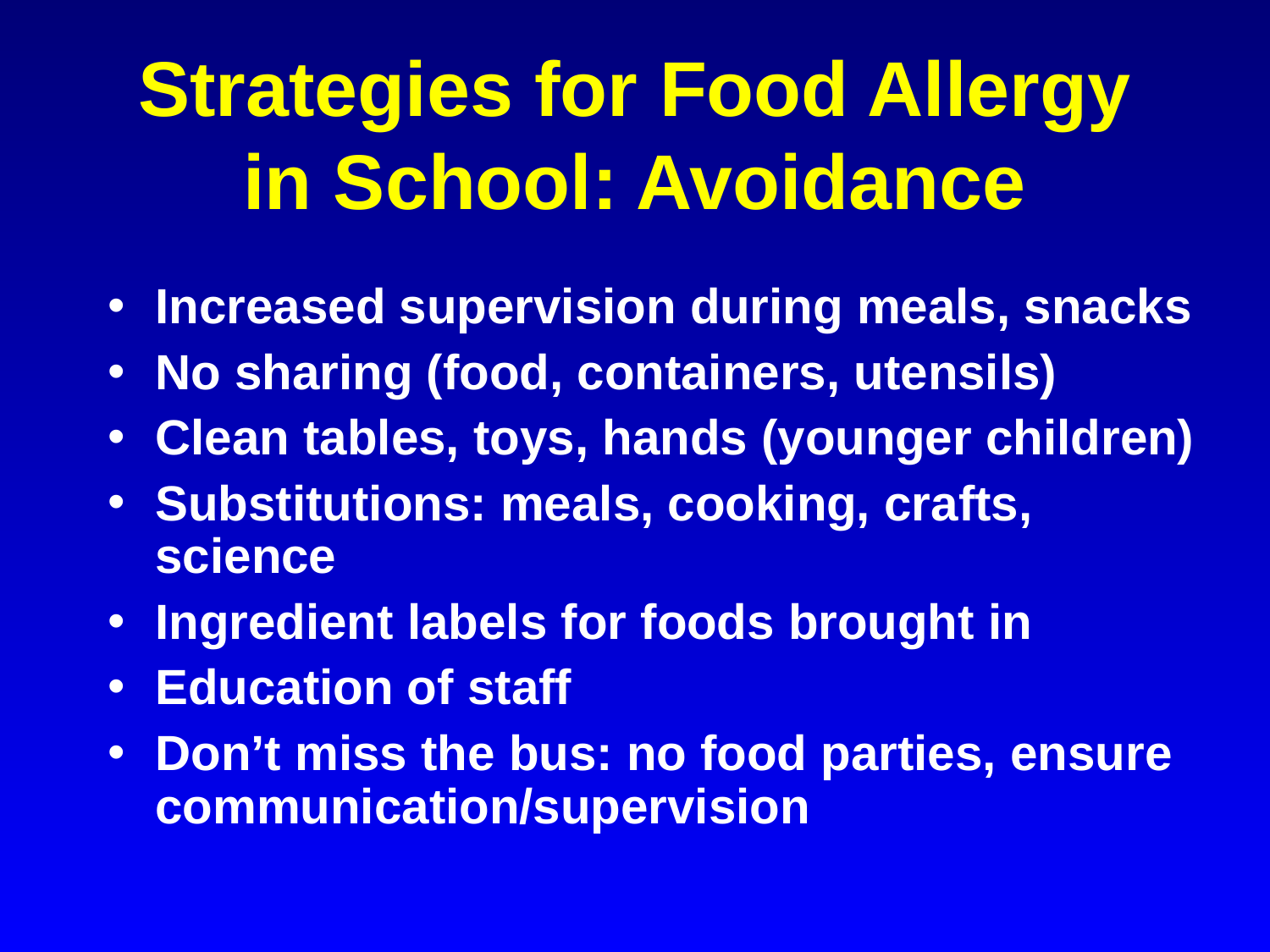

# Strategies for Food Allergy in School: Avoidance
Increased supervision during meals, snacks
No sharing (food, containers, utensils)
Clean tables, toys, hands (younger children)
Substitutions: meals, cooking, crafts, science
Ingredient labels for foods brought in
Education of staff
Don’t miss the bus: no food parties, ensure communication/supervision

## Slide 25
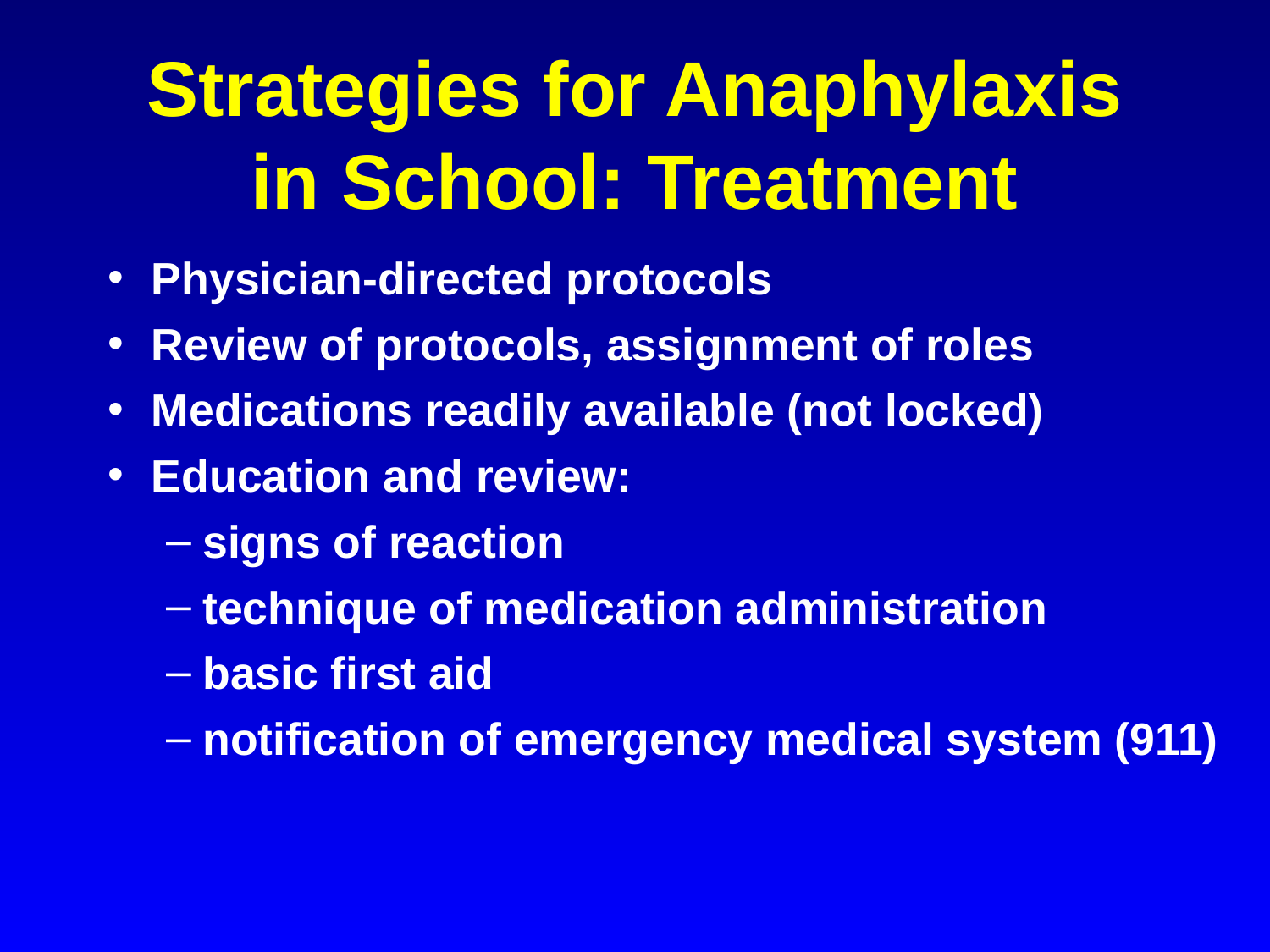

# Strategies for Anaphylaxis in School: Treatment
Physician-directed protocols
Review of protocols, assignment of roles
Medications readily available (not locked)
Education and review:
signs of reaction
technique of medication administration
basic first aid
notification of emergency medical system (911)

## Slide 26
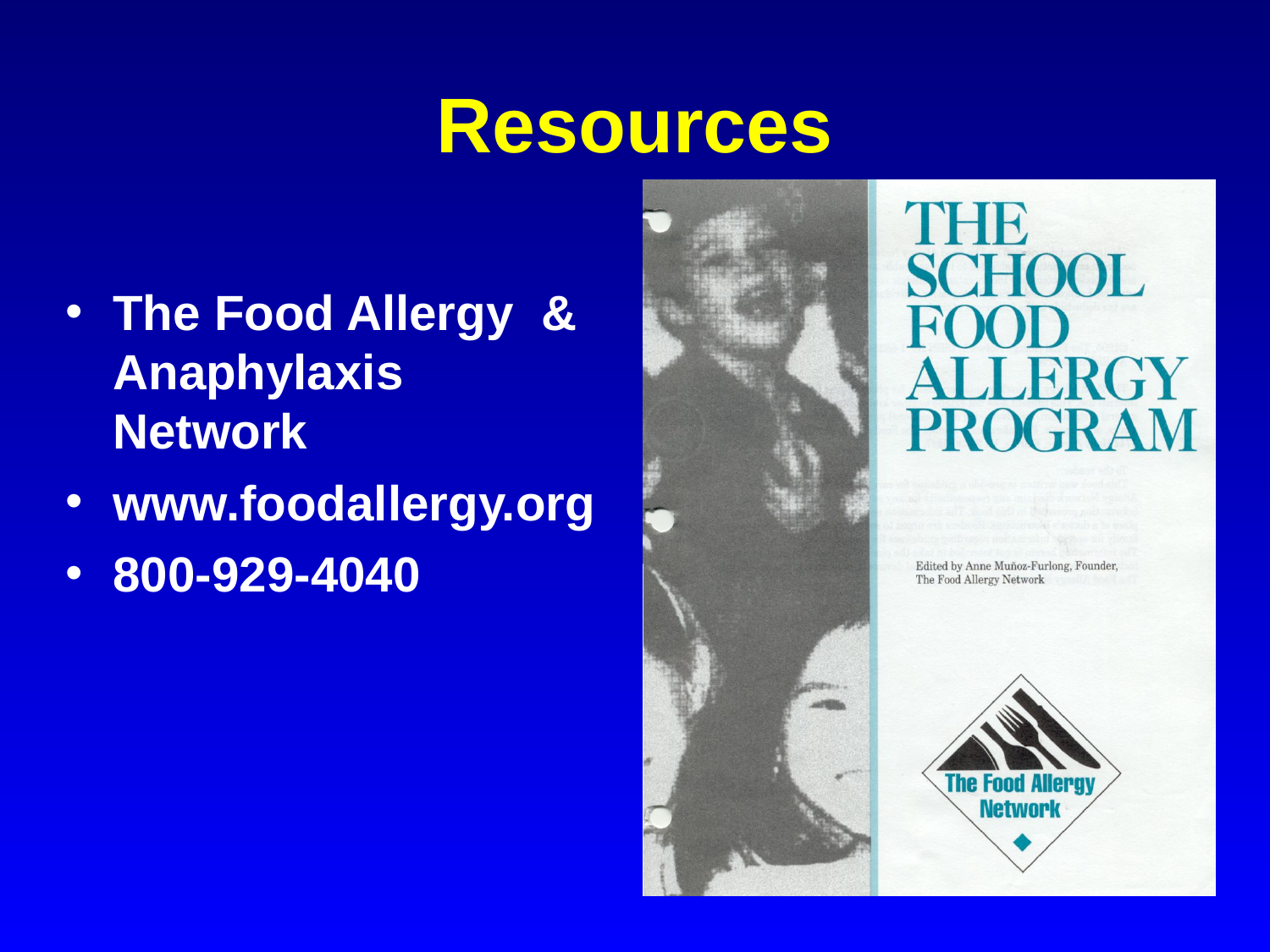

# Resources
The Food Allergy & Anaphylaxis Network
www.foodallergy.org
800-929-4040

## Slide 27
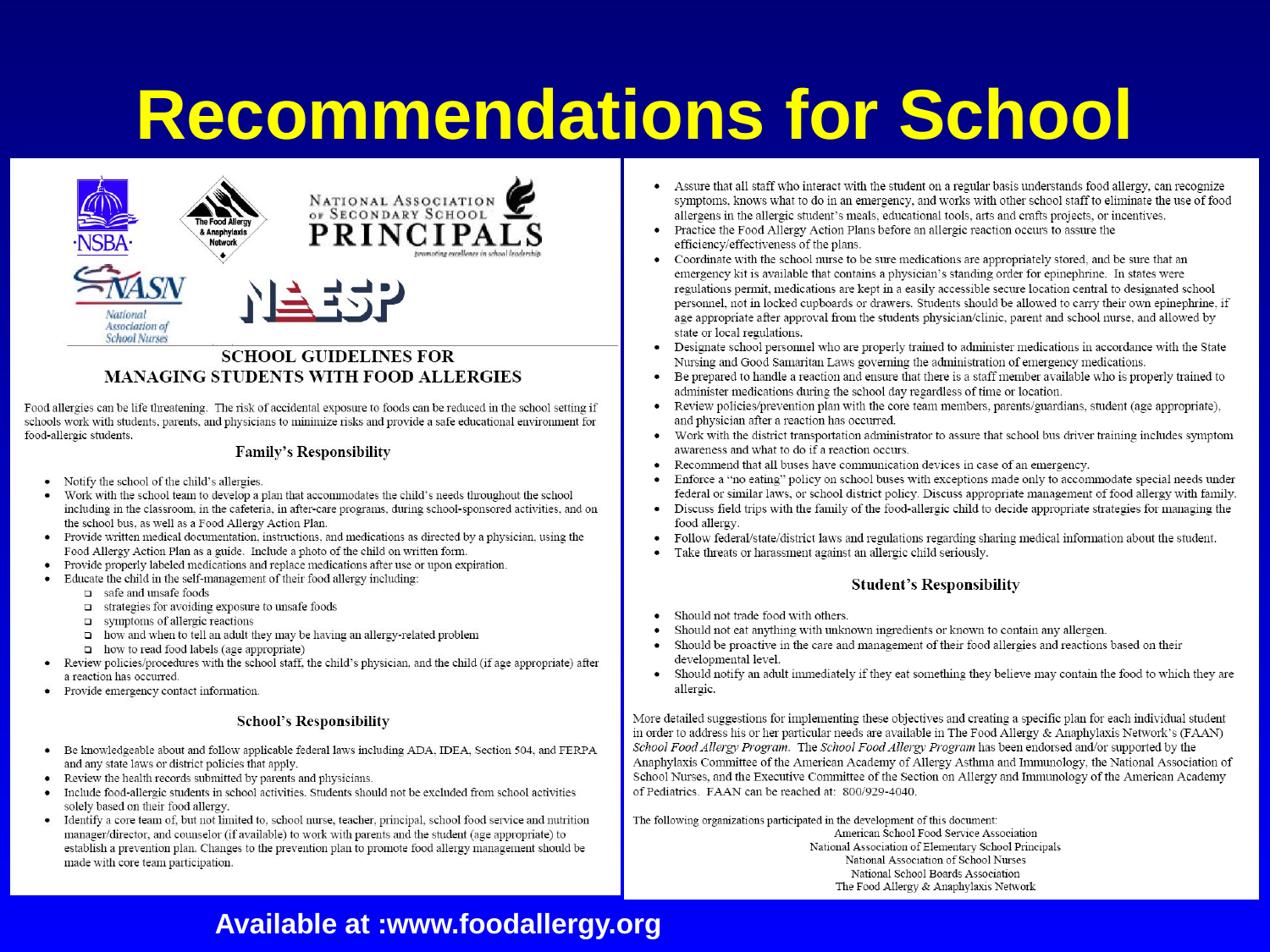

# Recommendations for School
Available at :www.foodallergy.org

## Slide 28
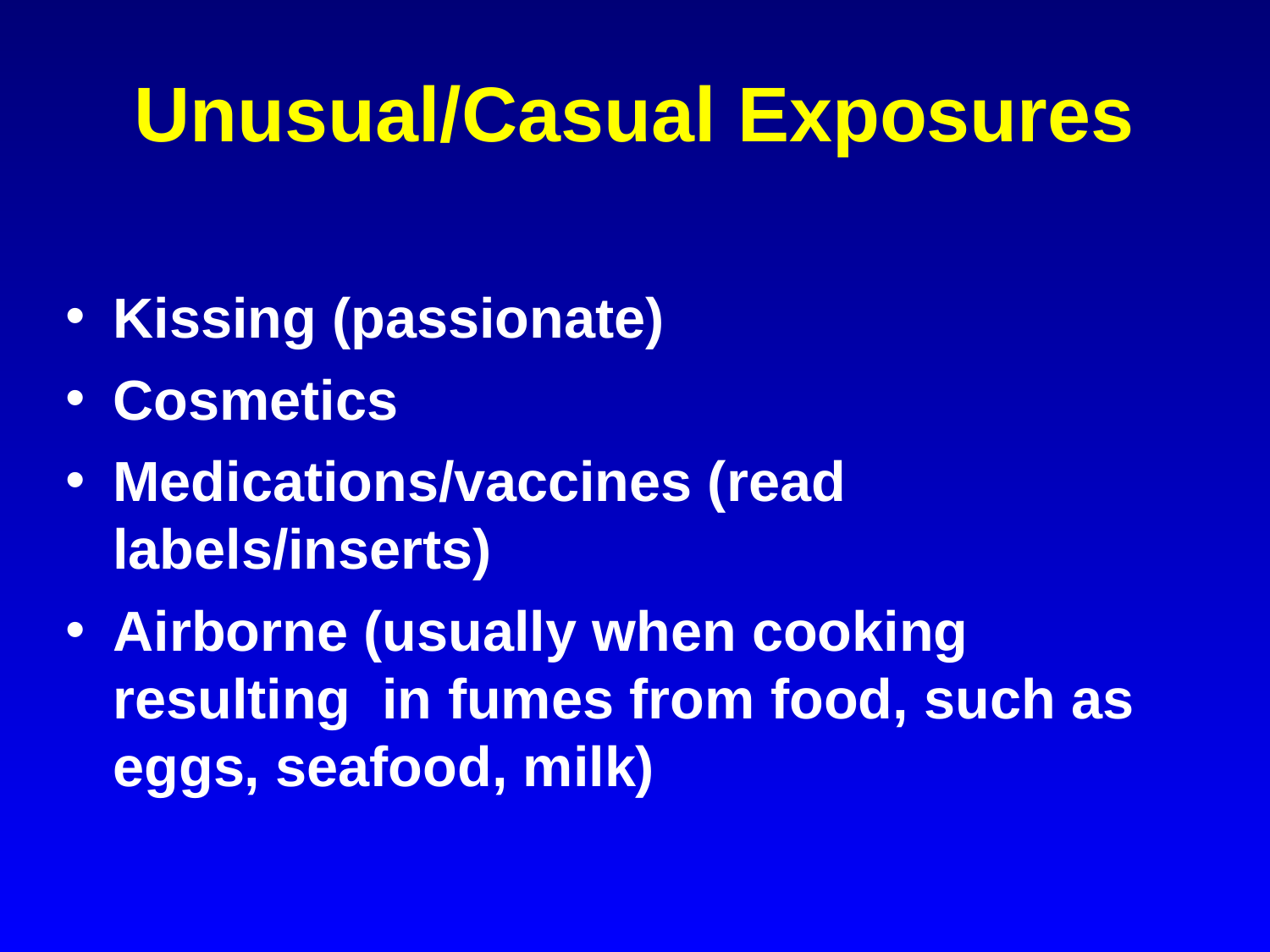

# Unusual/Casual Exposures
Kissing (passionate)
Cosmetics
Medications/vaccines (read labels/inserts)
Airborne (usually when cooking resulting in fumes from food, such as eggs, seafood, milk)

## Slide 29
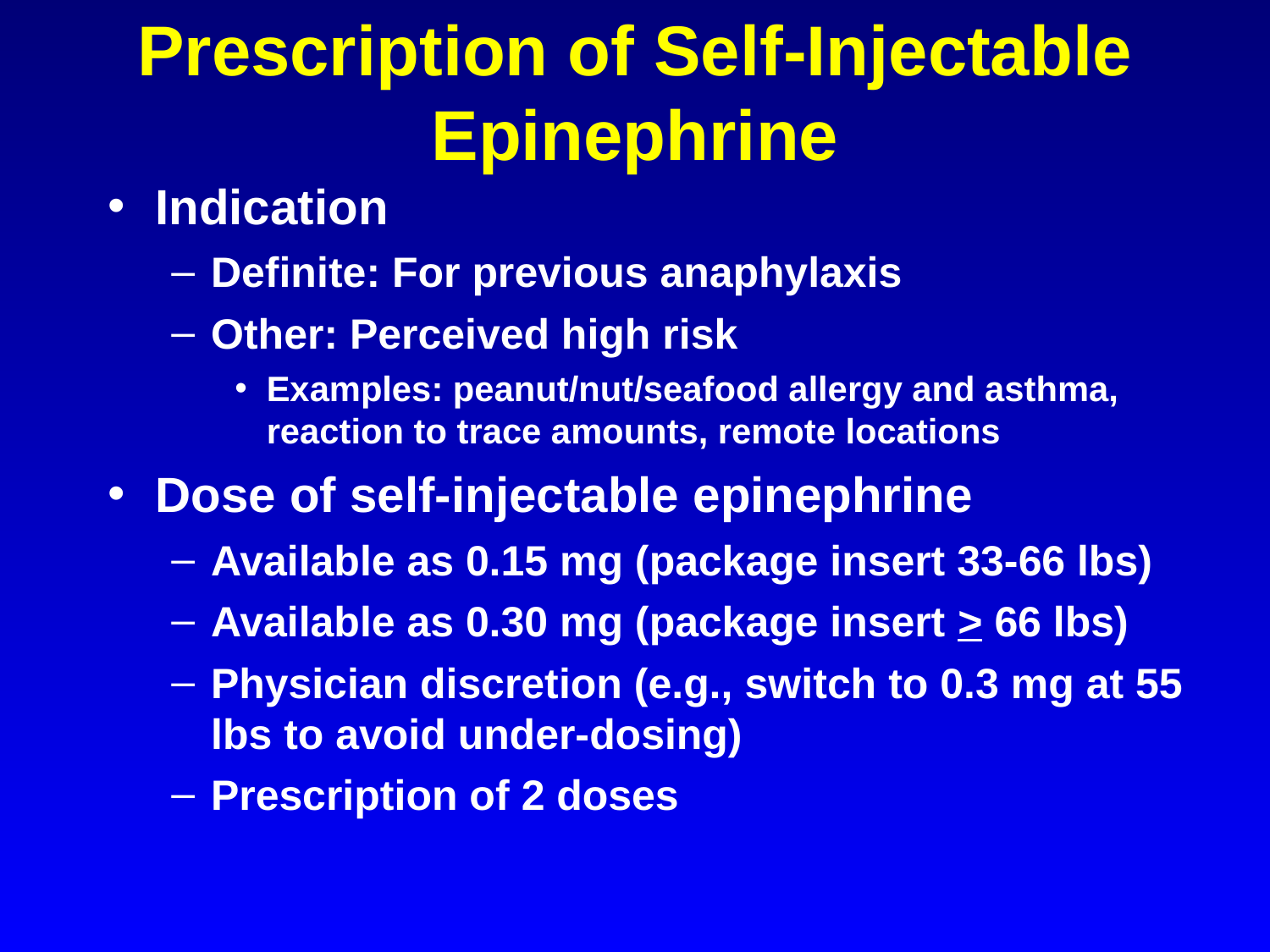

# Prescription of Self-Injectable Epinephrine
Indication
Definite: For previous anaphylaxis
Other: Perceived high risk
Examples: peanut/nut/seafood allergy and asthma, reaction to trace amounts, remote locations
Dose of self-injectable epinephrine
Available as 0.15 mg (package insert 33-66 lbs)
Available as 0.30 mg (package insert > 66 lbs)
Physician discretion (e.g., switch to 0.3 mg at 55 lbs to avoid under-dosing)
Prescription of 2 doses

## Slide 30
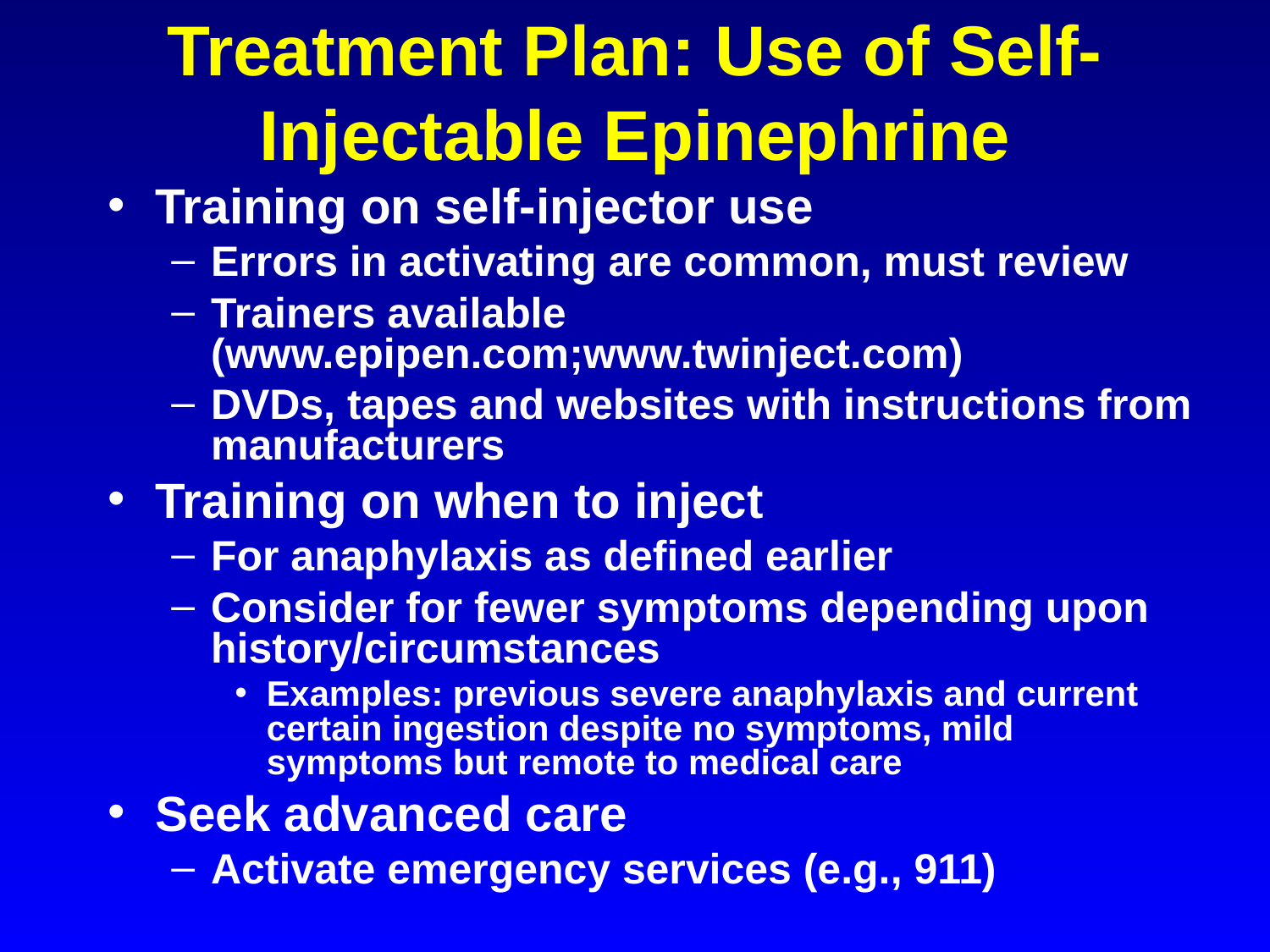

# Treatment Plan: Use of Self-Injectable Epinephrine
Training on self-injector use
Errors in activating are common, must review
Trainers available (www.epipen.com;www.twinject.com)
DVDs, tapes and websites with instructions from manufacturers
Training on when to inject
For anaphylaxis as defined earlier
Consider for fewer symptoms depending upon history/circumstances
Examples: previous severe anaphylaxis and current certain ingestion despite no symptoms, mild symptoms but remote to medical care
Seek advanced care
Activate emergency services (e.g., 911)

## Slide 31
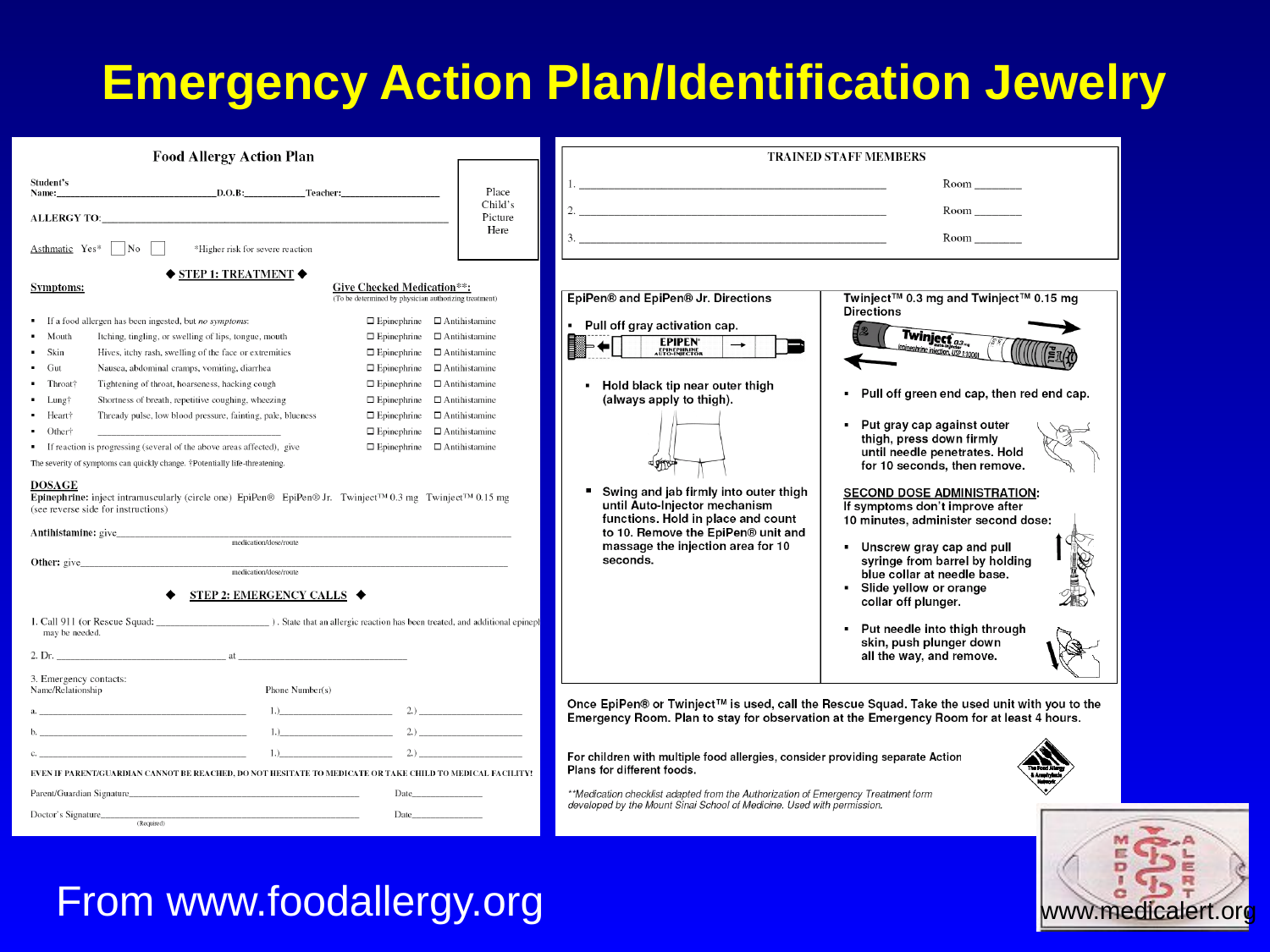

# Emergency Action Plan/Identification Jewelry
From www.foodallergy.org
www.medicalert.org

## Slide 32
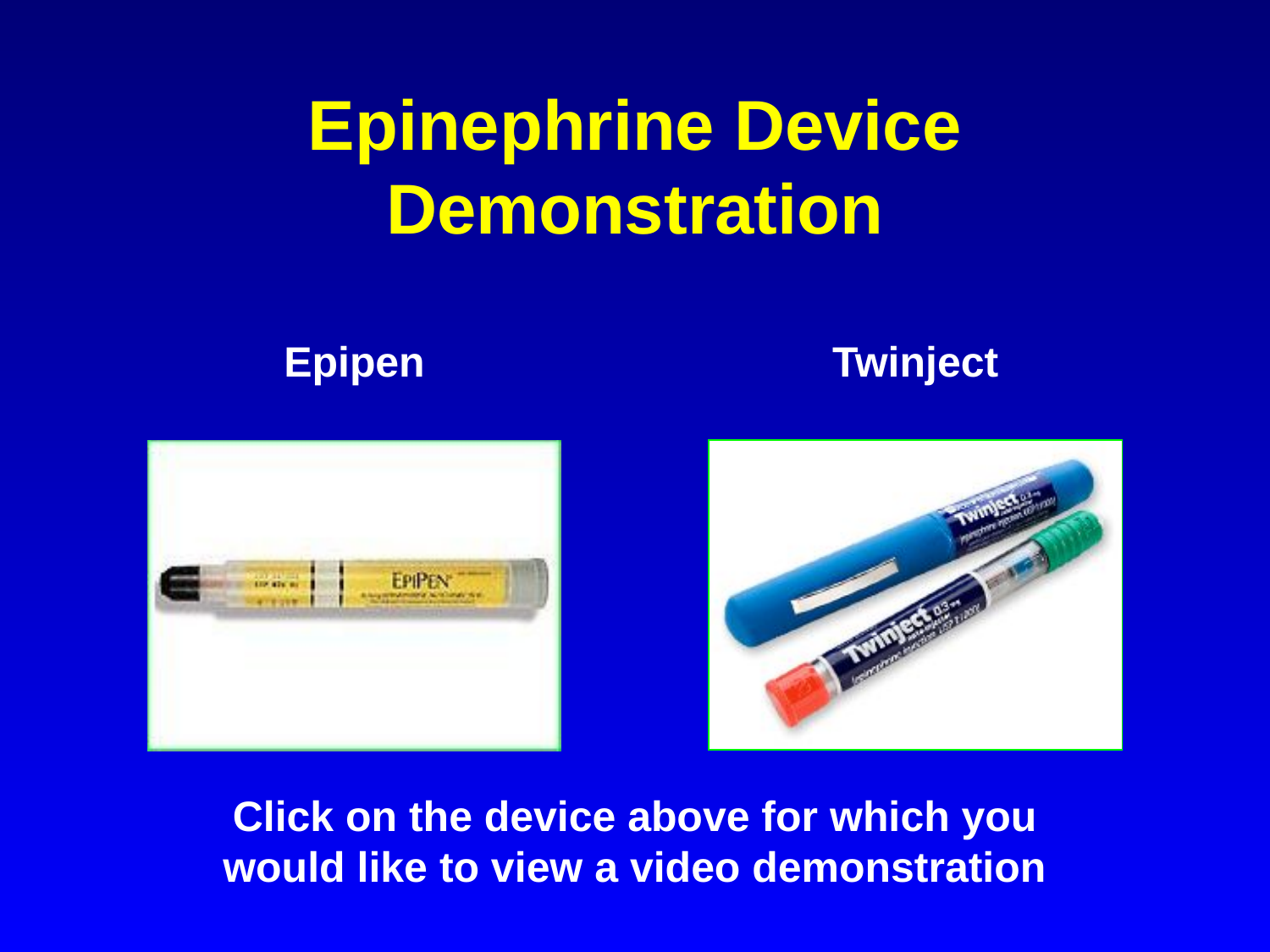

# Epinephrine Device Demonstration
Epipen
Twinject
Click on the device above for which you would like to view a video demonstration

## Slide 33
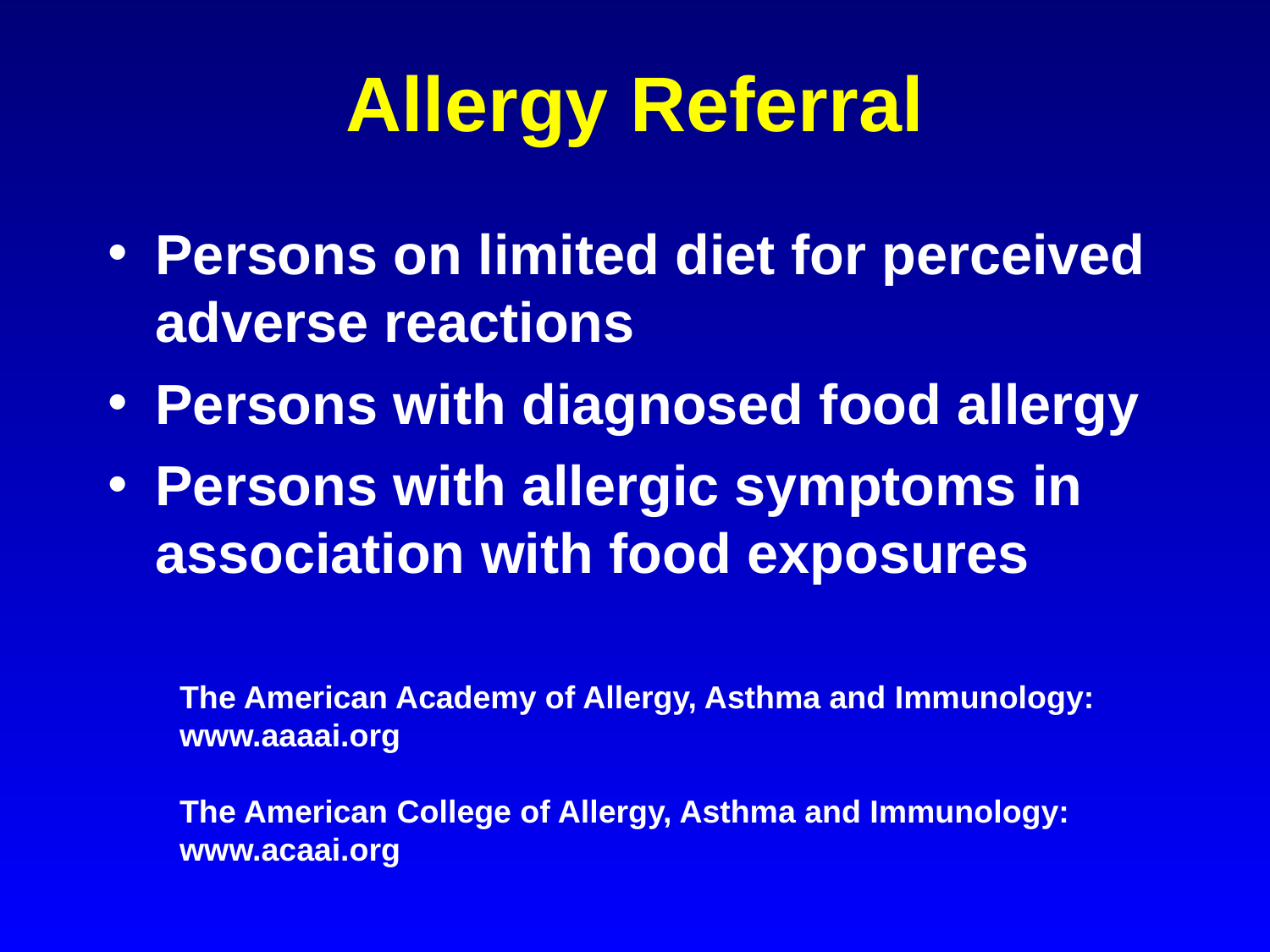

# Allergy Referral
Persons on limited diet for perceived adverse reactions
Persons with diagnosed food allergy
Persons with allergic symptoms in association with food exposures
The American Academy of Allergy, Asthma and Immunology: www.aaaai.org
The American College of Allergy, Asthma and Immunology: www.acaai.org

## Slide 34
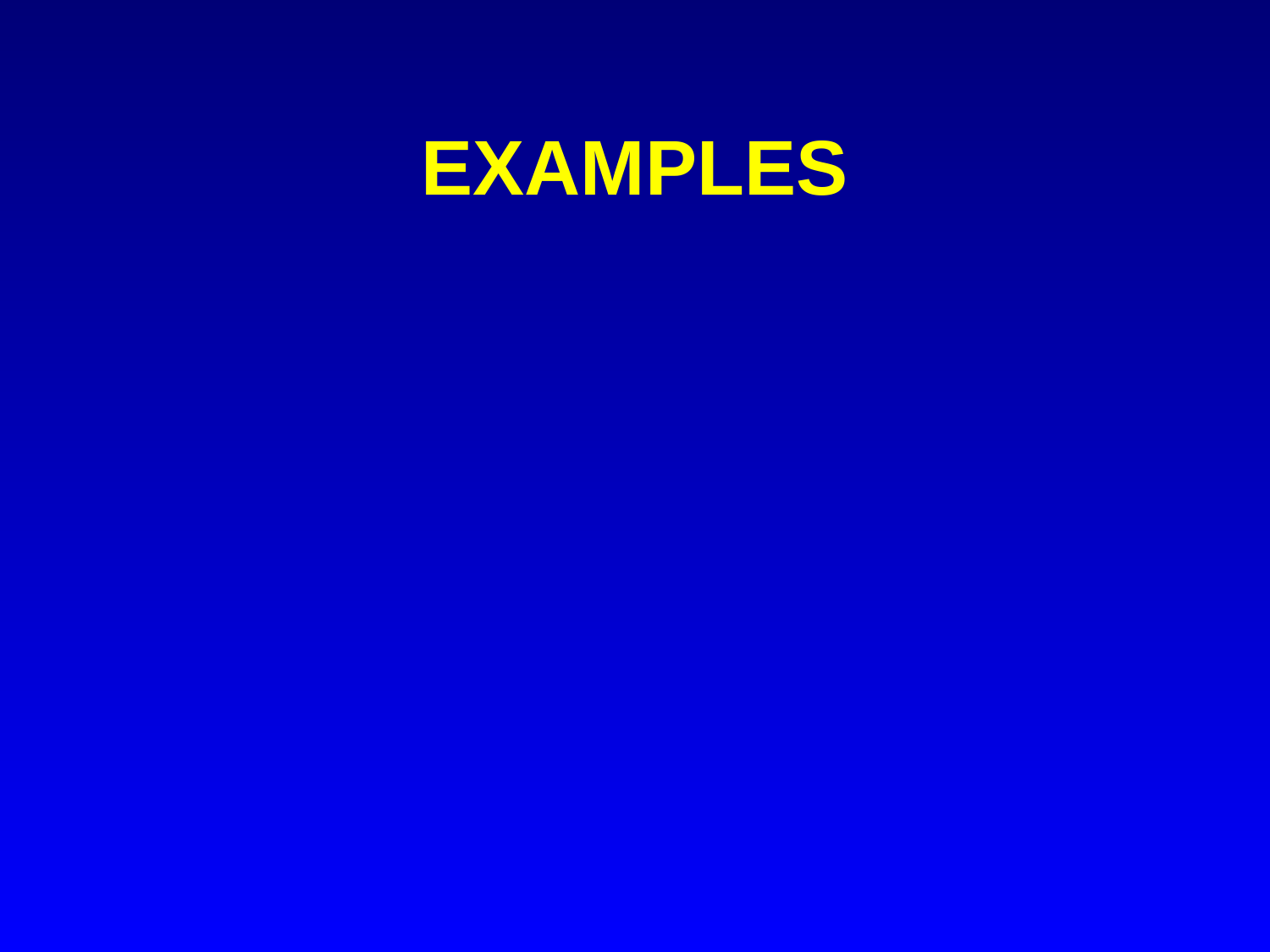

# EXAMPLES

## Slide 35
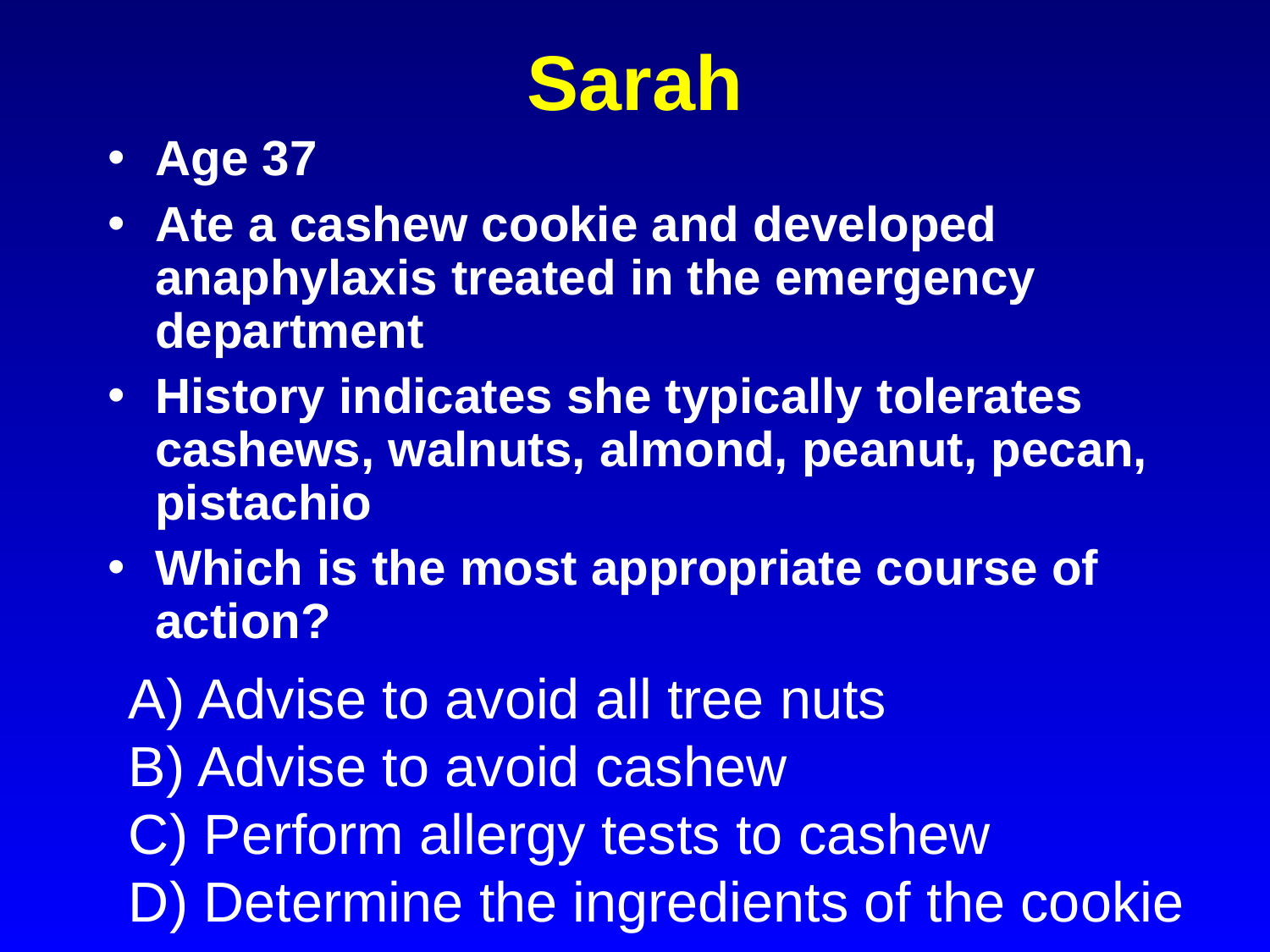

# Sarah
Age 37
Ate a cashew cookie and developed anaphylaxis treated in the emergency department
History indicates she typically tolerates cashews, walnuts, almond, peanut, pecan, pistachio
Which is the most appropriate course of action?
A) Advise to avoid all tree nuts
B) Advise to avoid cashew
C) Perform allergy tests to cashew
D) Determine the ingredients of the cookie

## Slide 36
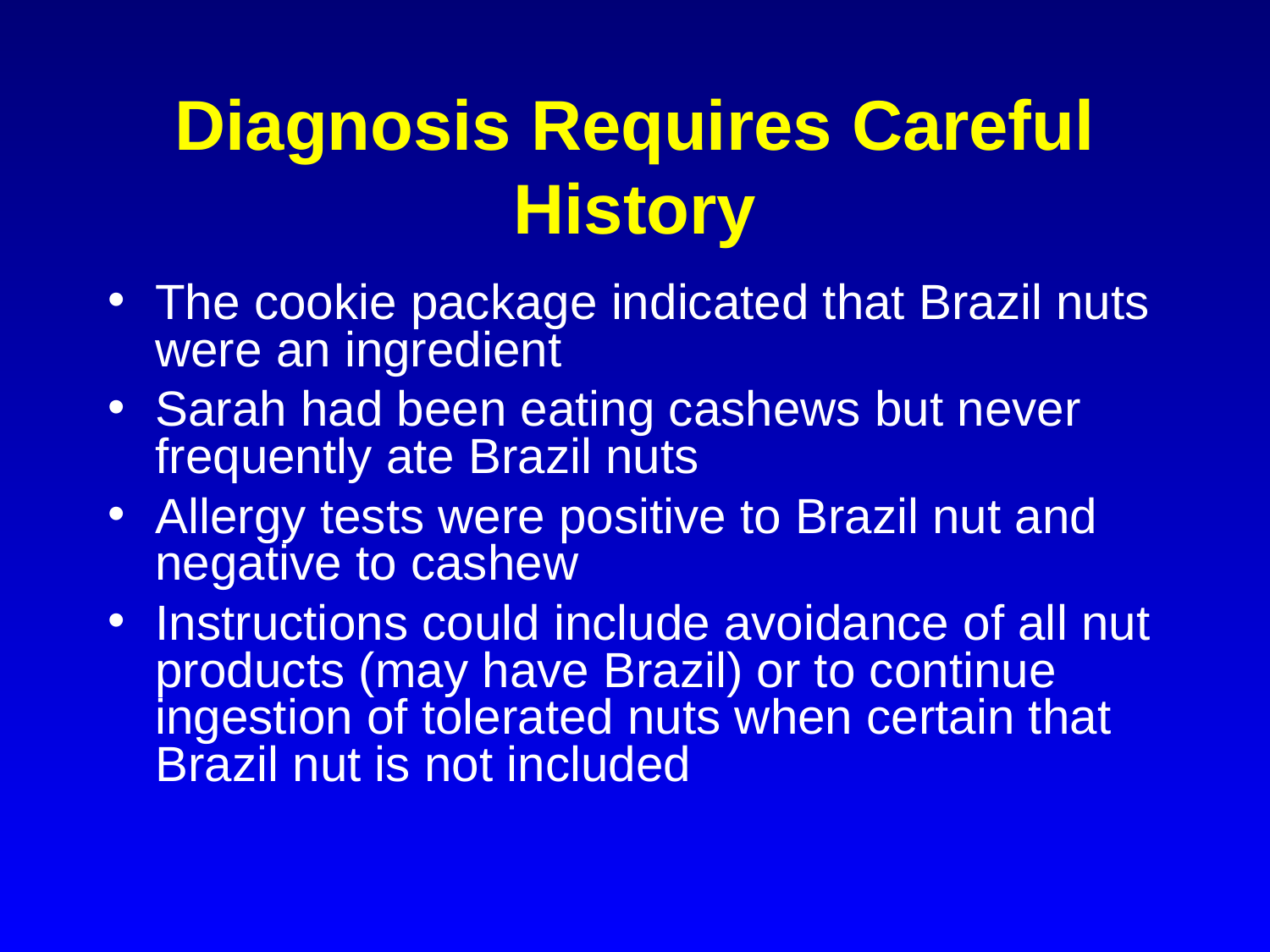

# Diagnosis Requires Careful History
The cookie package indicated that Brazil nuts were an ingredient
Sarah had been eating cashews but never frequently ate Brazil nuts
Allergy tests were positive to Brazil nut and negative to cashew
Instructions could include avoidance of all nut products (may have Brazil) or to continue ingestion of tolerated nuts when certain that Brazil nut is not included

## Slide 37
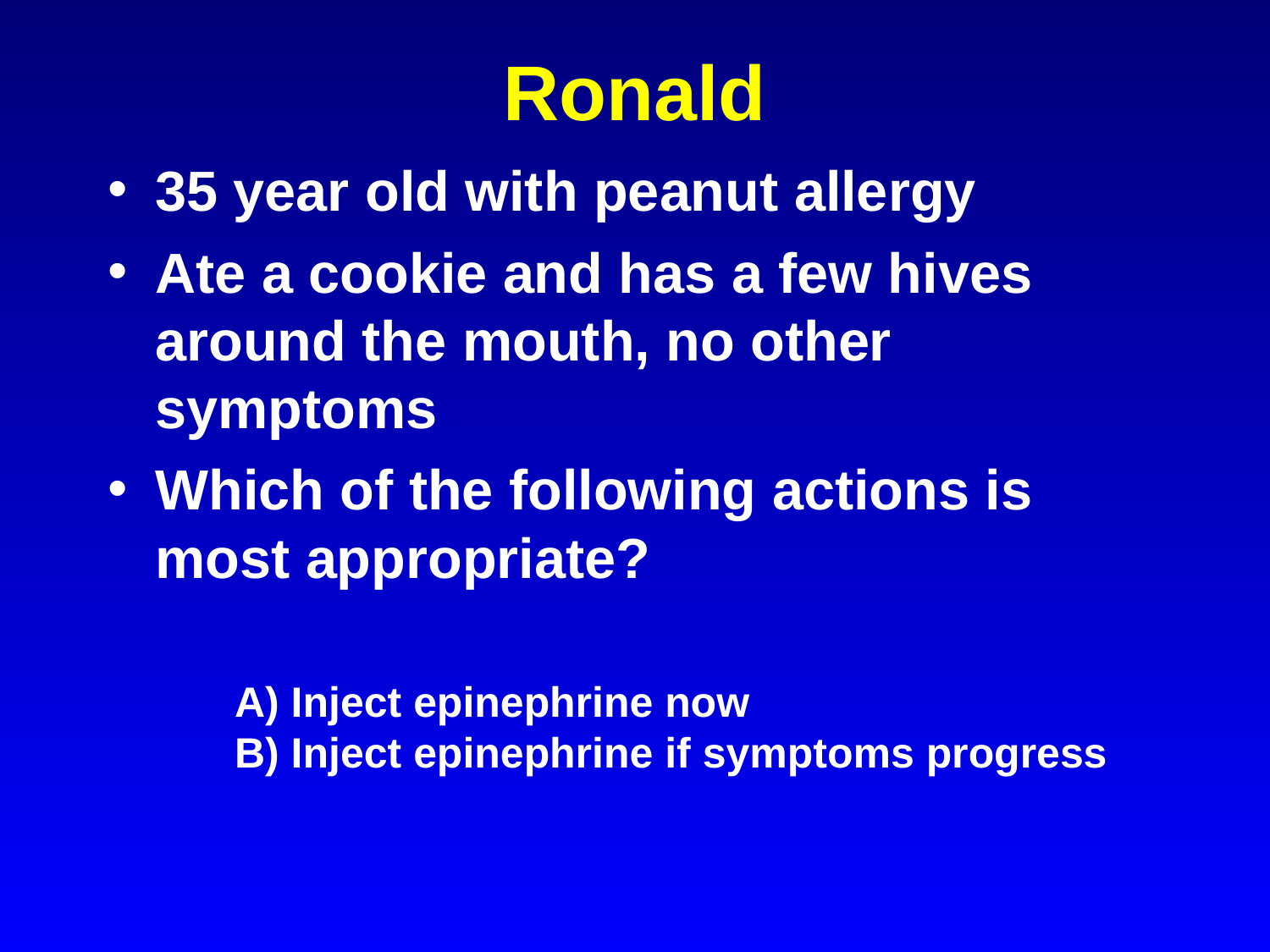

# Ronald
35 year old with peanut allergy
Ate a cookie and has a few hives around the mouth, no other symptoms
Which of the following actions is most appropriate?
A) Inject epinephrine now
B) Inject epinephrine if symptoms progress

## Slide 38
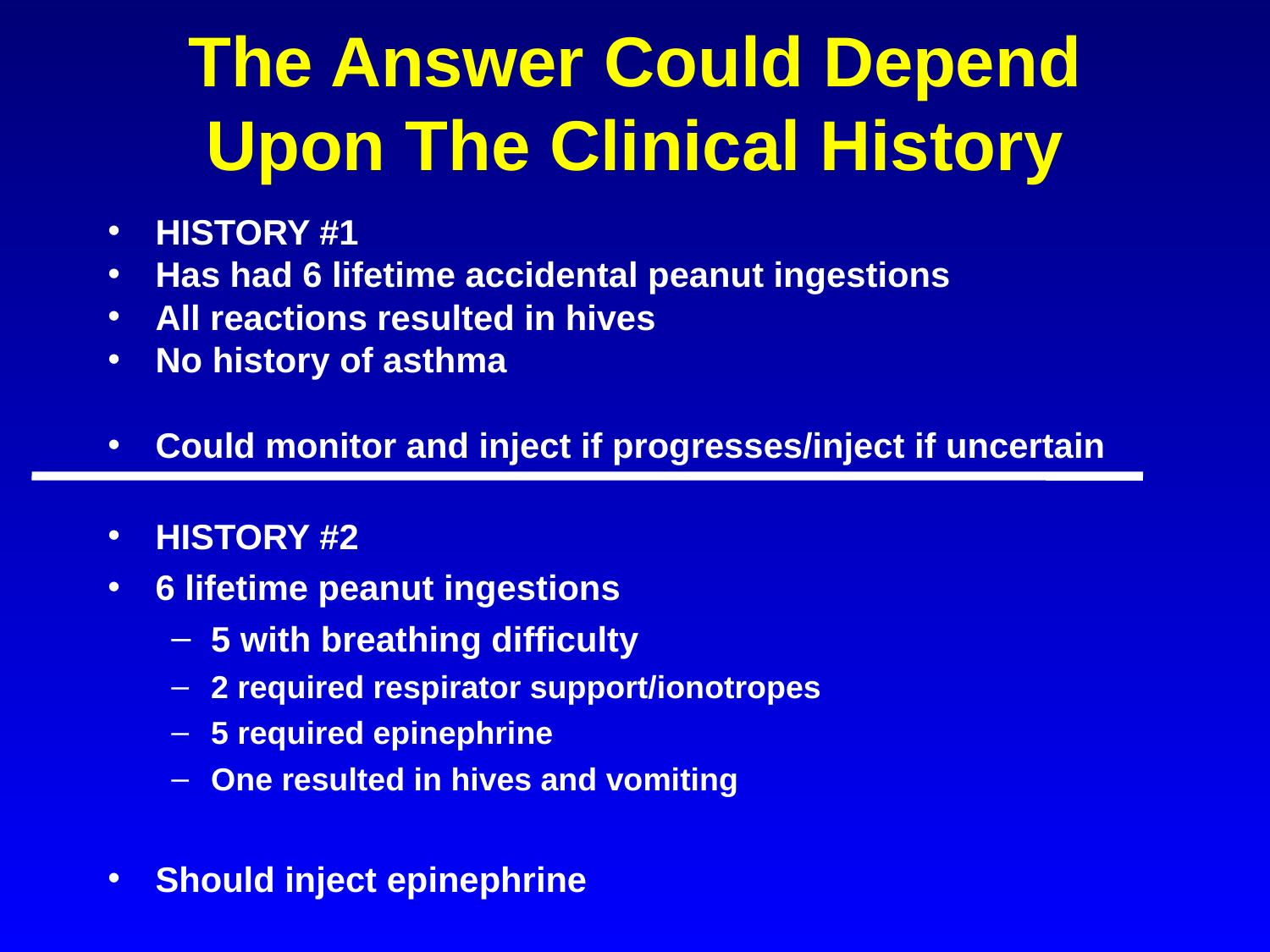

# The Answer Could Depend Upon The Clinical History
HISTORY #1
Has had 6 lifetime accidental peanut ingestions
All reactions resulted in hives
No history of asthma
Could monitor and inject if progresses/inject if uncertain
HISTORY #2
6 lifetime peanut ingestions
5 with breathing difficulty
2 required respirator support/ionotropes
5 required epinephrine
One resulted in hives and vomiting
Should inject epinephrine

## Slide 39
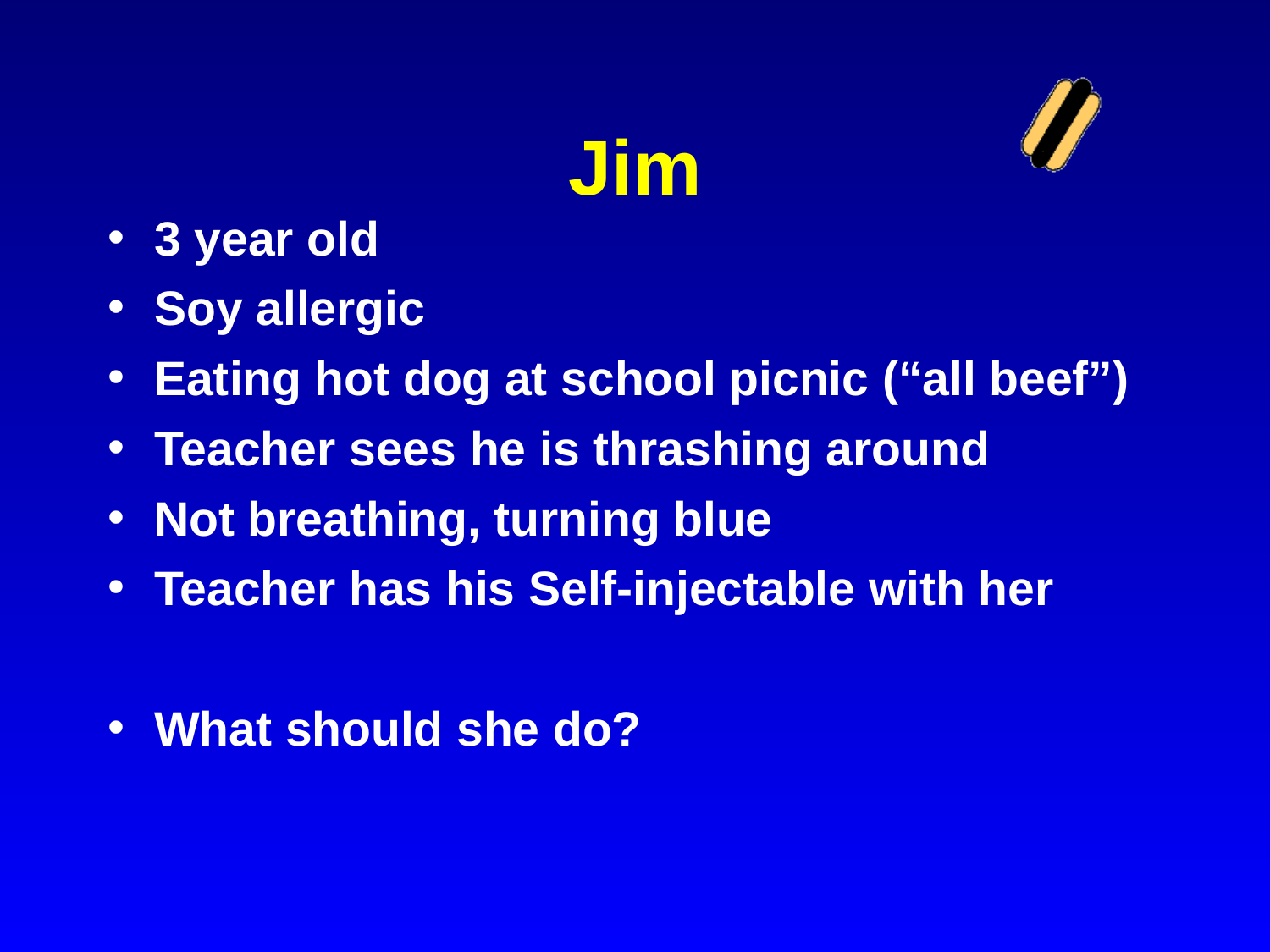

# Jim
3 year old
Soy allergic
Eating hot dog at school picnic (“all beef”)
Teacher sees he is thrashing around
Not breathing, turning blue
Teacher has his Self-injectable with her
What should she do?

## Slide 40
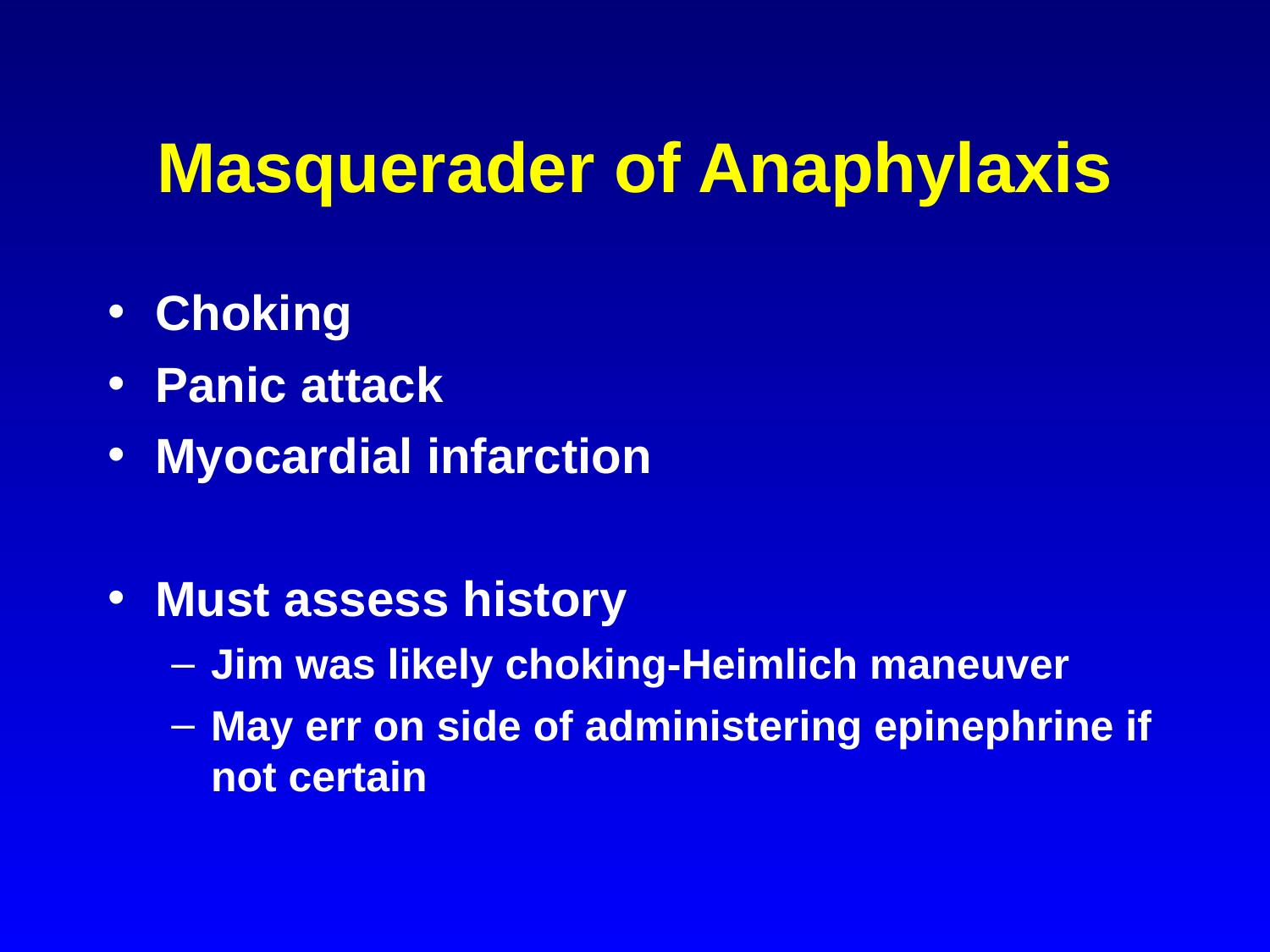

# Masquerader of Anaphylaxis
Choking
Panic attack
Myocardial infarction
Must assess history
Jim was likely choking-Heimlich maneuver
May err on side of administering epinephrine if not certain

## Slide 41
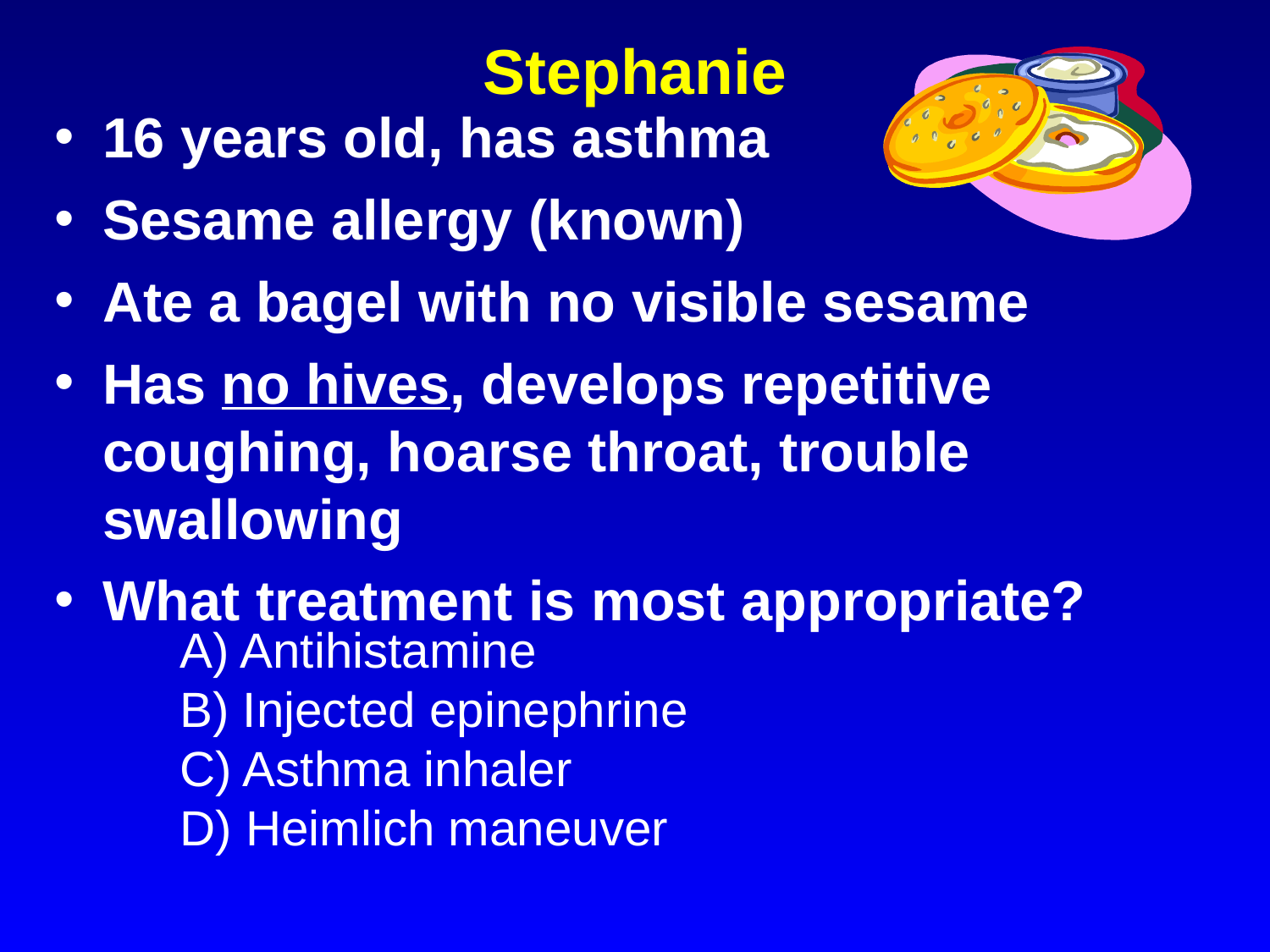

# Stephanie
16 years old, has asthma
Sesame allergy (known)
Ate a bagel with no visible sesame
Has no hives, develops repetitive coughing, hoarse throat, trouble swallowing
What treatment is most appropriate?
A) Antihistamine
B) Injected epinephrine
C) Asthma inhaler
D) Heimlich maneuver

## Slide 42
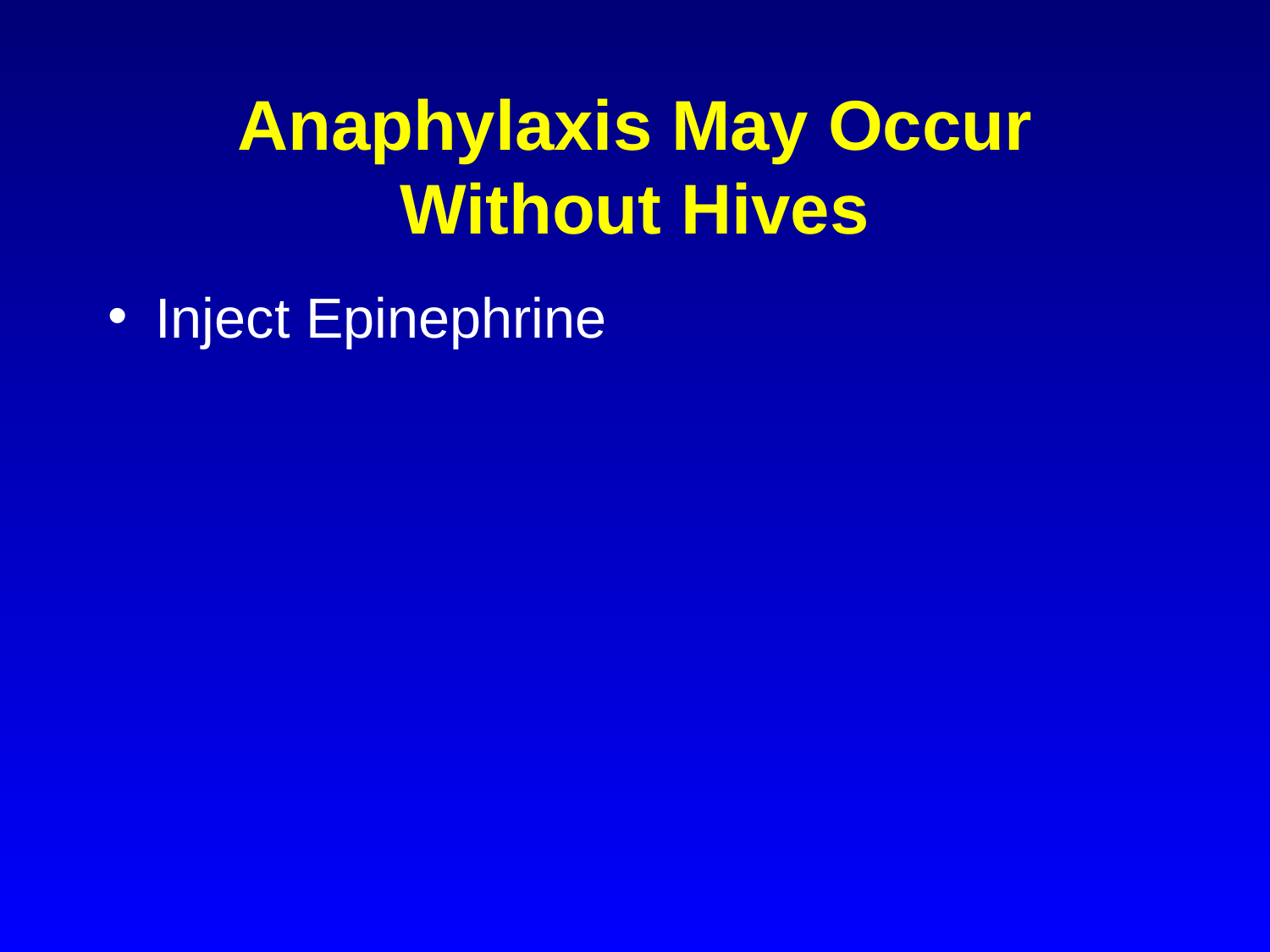

# Anaphylaxis May Occur Without Hives
Inject Epinephrine

## Slide 43
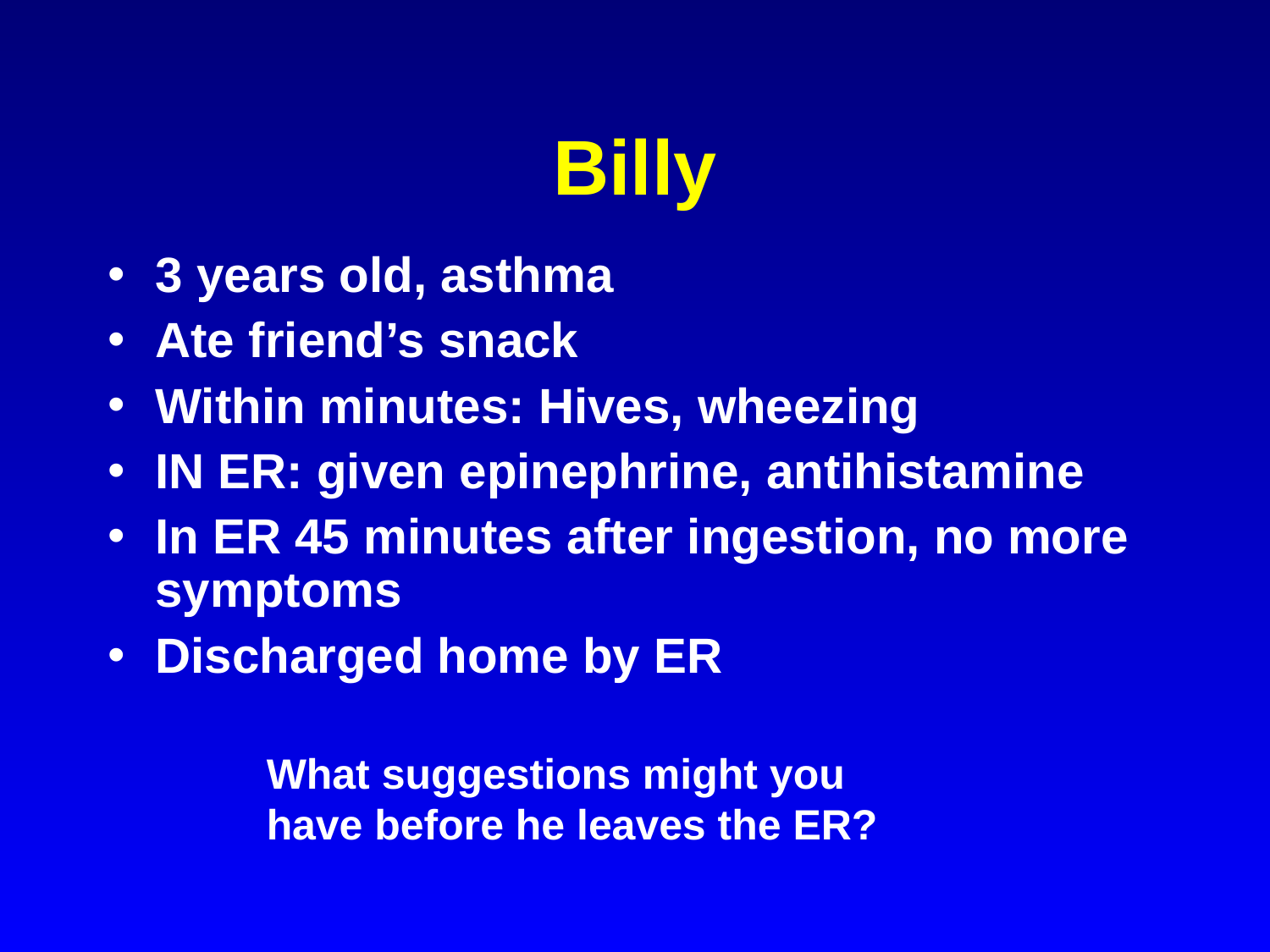

# Billy
3 years old, asthma
Ate friend’s snack
Within minutes: Hives, wheezing
IN ER: given epinephrine, antihistamine
In ER 45 minutes after ingestion, no more symptoms
Discharged home by ER
What suggestions might you have before he leaves the ER?

## Slide 44
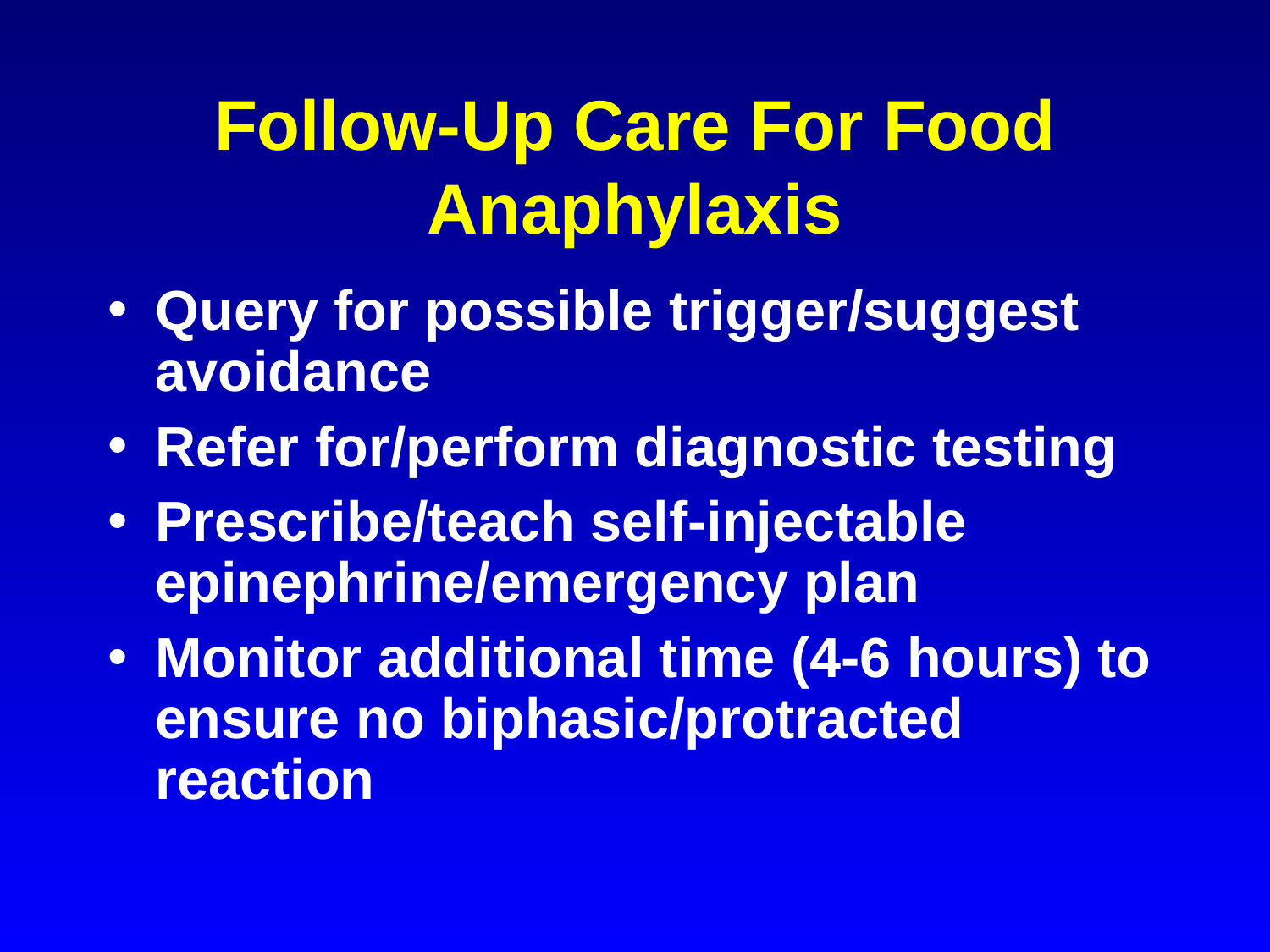

# Follow-Up Care For Food Anaphylaxis
Query for possible trigger/suggest avoidance
Refer for/perform diagnostic testing
Prescribe/teach self-injectable epinephrine/emergency plan
Monitor additional time (4-6 hours) to ensure no biphasic/protracted reaction

## Slide 45
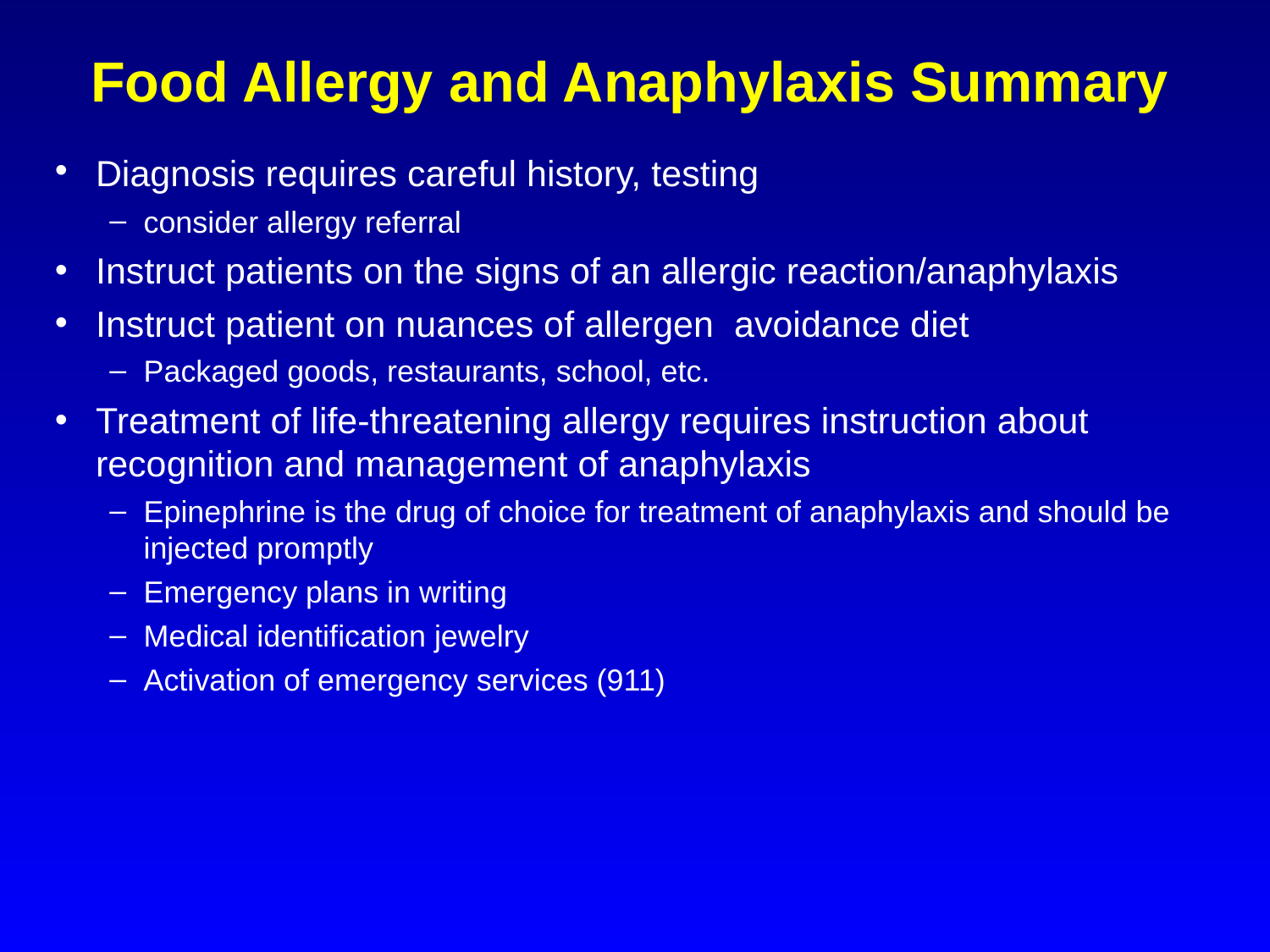

# Food Allergy and Anaphylaxis Summary
Diagnosis requires careful history, testing
consider allergy referral
Instruct patients on the signs of an allergic reaction/anaphylaxis
Instruct patient on nuances of allergen avoidance diet
Packaged goods, restaurants, school, etc.
Treatment of life-threatening allergy requires instruction about recognition and management of anaphylaxis
Epinephrine is the drug of choice for treatment of anaphylaxis and should be injected promptly
Emergency plans in writing
Medical identification jewelry
Activation of emergency services (911)

## Slide 46
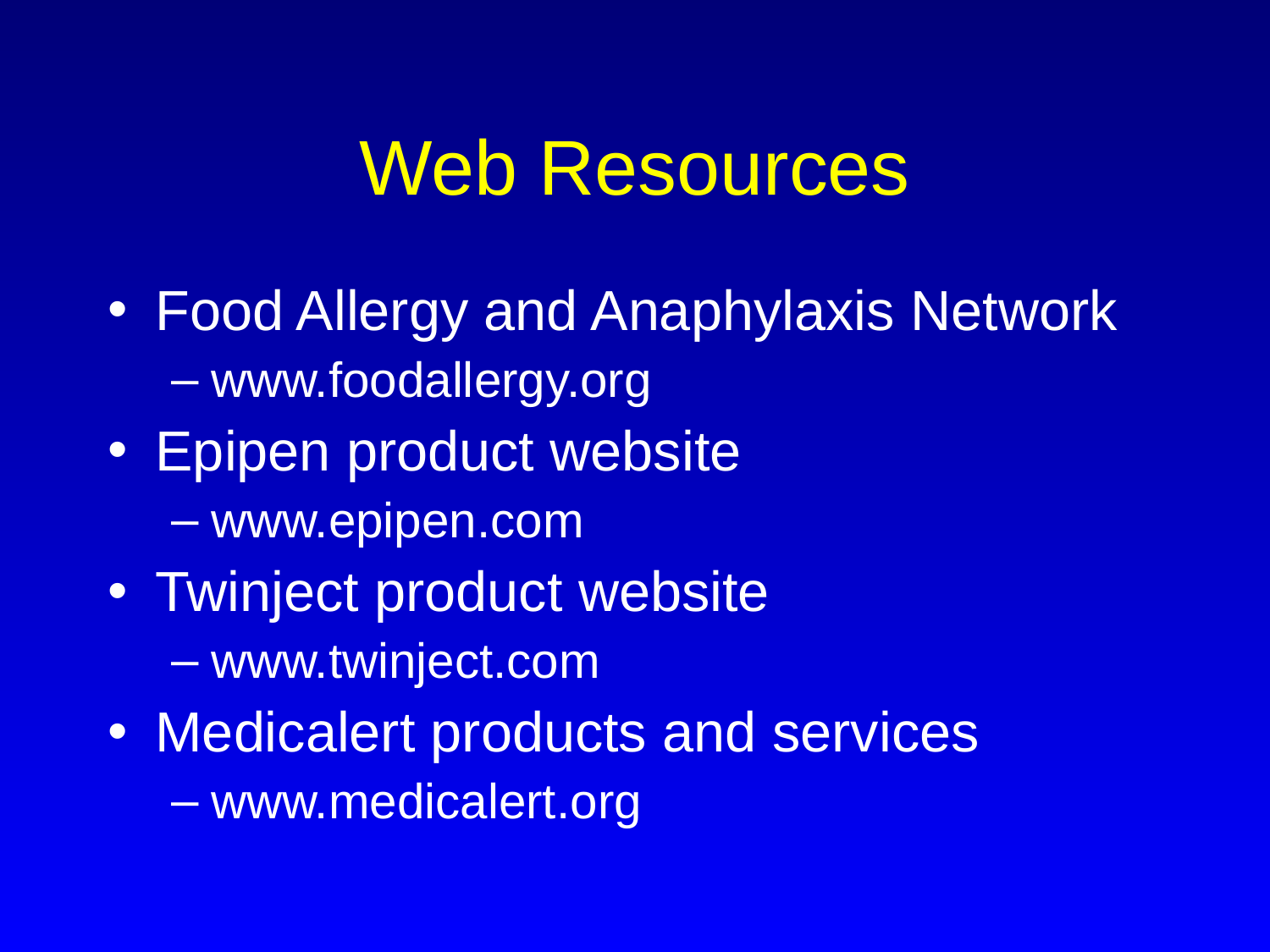

# Web Resources
Food Allergy and Anaphylaxis Network
www.foodallergy.org
Epipen product website
www.epipen.com
Twinject product website
www.twinject.com
Medicalert products and services
www.medicalert.org

## Slide 47
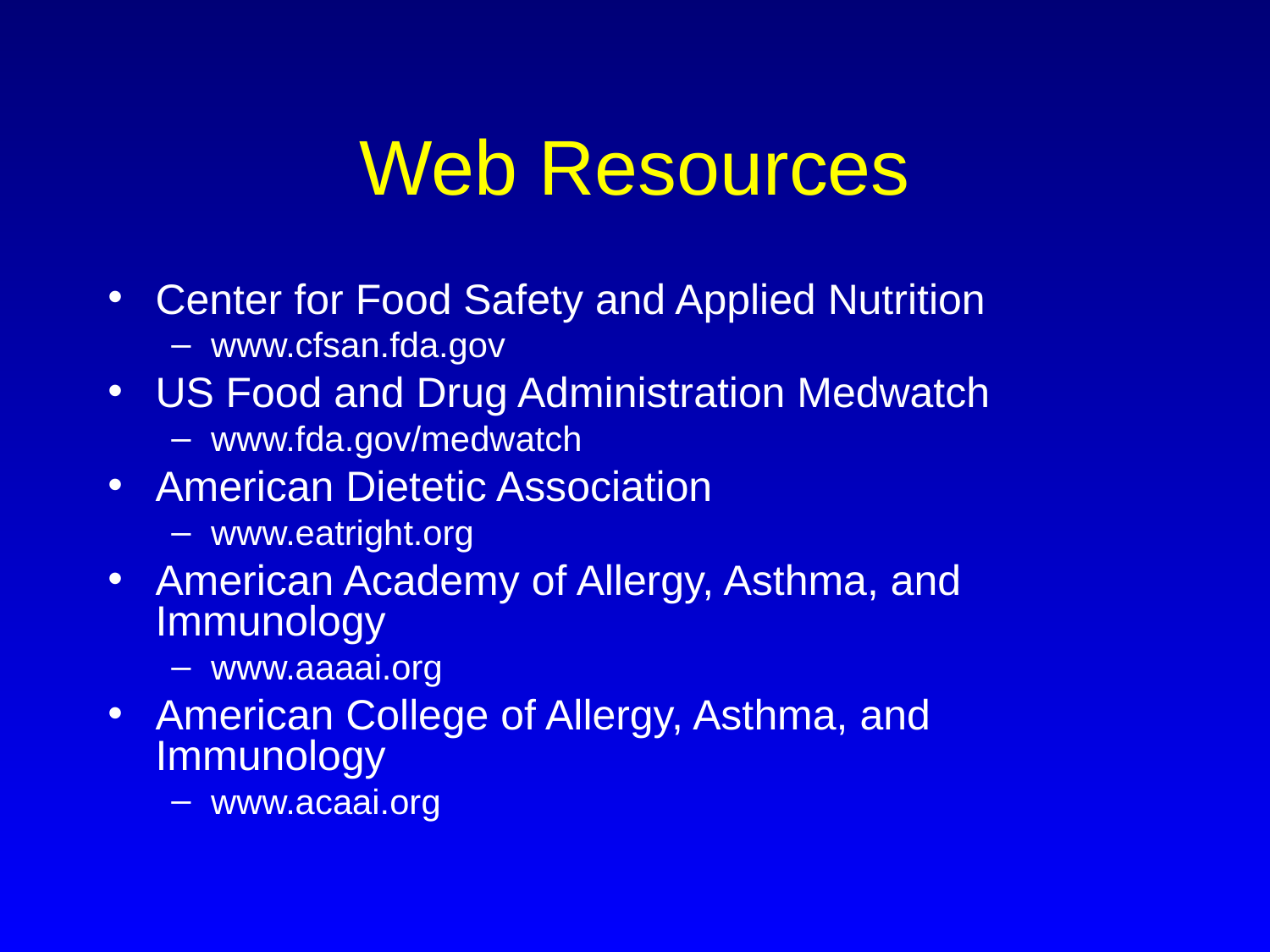

# Web Resources
Center for Food Safety and Applied Nutrition
www.cfsan.fda.gov
US Food and Drug Administration Medwatch
www.fda.gov/medwatch
American Dietetic Association
www.eatright.org
American Academy of Allergy, Asthma, and Immunology
www.aaaai.org
American College of Allergy, Asthma, and Immunology
www.acaai.org
